# Two-Phase Bactericidal Mechanism of Silver Nanoparticles against *Burkholderia pseudomallei*

**DOI:** 10.1371/journal.pone.0168098

**Published:** 2016-12-15

**Authors:** Pawinee Siritongsuk, Nuttaya Hongsing, Saengrawee Thammawithan, Sakda Daduang, Sompong Klaynongsruang, Apichai Tuanyok, Rina Patramanon

**Affiliations:** 1 Department of Biochemistry, Faculty of Science, Khon Kaen University, Khon Kaen, Thailand; 2 Protein and Proteomics Research Center for Commercial and Industrial Purposes, Khon Kaen University, Khon Kaen, Thailand; 3 Faculty of Pharmaceutical Sciences, Khon Kaen University, Khon Kaen, Thailand; 4 College of Veterinary Medicine and Emerging Pathogens Institute, University of Florida, Gainesville, Florida, United States of America; Tulane University School of Medicine, UNITED STATES

## Abstract

Silver nanoparticles (AgNPs) have a strong antimicrobial activity against a variety of pathogenic bacteria. The killing mechanism of AgNPs involves direct physical membrane destruction and subsequent molecular damage from both AgNPs and released Ag^+^. *Burkholderia pseudomallei* is the causative agent of melioidosis, an endemic infectious disease primarily found in northern Australia and Southeast Asia. *B*. *pseudomallei* is intrinsically resistant to most common antibiotics. In this study, the antimicrobial activity and mechanism of AgNPs (10–20 nm) against *B*. *pseudomallei* were investigated. The MIC and MBC for nine *B*. *pseudomallei* strains ranged from 32–48 μg/mL and 96–128 μg/mL, respectively. Concentrations of AgNPs less than 256 μg/mL were not toxic to human red blood cells. AgNPs exhibited a two-phase mechanism: cell death induction and ROS induction. The first phase was a rapid killing step within 5 min, causing the direct damage of the cytoplasmic membrane of the bacterial cells, as observed by a time-kill assay and fluorescence microscopy. During the period of 5–30 min, the cell surface charge was rapidly neutralized from -8.73 and -7.74 to 2.85 and 2.94 mV in two isolates of *B*. *pseudomallei*, as revealed by zeta potential measurement. Energy-dispersive X-ray (EDX) spectroscopy showed the silver element deposited on the bacterial membrane, and TEM micrographs of the AgNP-treated *B*. *pseudomallei* cells showed severe membrane damage and cytosolic leakage at 1/5 MIC and cell bursting at MBC. During the killing effect the released Ag^+^ from AgNPs was only 3.9% from the starting AgNPs concentration as observed with ICP-OES experiment. In the second phase, the ROS induction occurred 1–4 hr after the AgNP treatment. Altogether, we provide direct kinetic evidence of the AgNPs killing mechanism, by which cell death is separable from the ROS induction and AgNPs mainly contributes in the killing action. AgNPs may be considered a potential candidate to develop a novel alternative agent for melioidosis treatment with fast action.

## Introduction

Developments in nanotechnology have enabled us to utilize the properties of metals in the size range of 1–100 nm, a.k.a. nanoparticles, in various applications. Newly developed properties of metal nanoparticles have allowed us to explore their applicability in biomarkers, diagnostics, antimicrobial agents and nano-drugs for defense against various infectious diseases [1, 2]. Silver has a strong antimicrobial potential and has been used as a cleaning agent since ancient times. AgNPs has been shown to have strong growth inhibitory and cytotoxic effects against bacteria, fungi, and viruses [3–5]. AgNPs in the size rage of 10–100 nm have shown strong bactericidal potential against a broad spectrum of antibiotic-resistant bacterial strains [6, 7]. In addition, AgNPs exhibit high toxicity to microorganisms but low toxicity to mammalian cells [8]. The antibacterial activity and mode of action of AgNPs are dependent on their size, shape, dispersion, concentration, dose, agglomeration and dissolution rate [2, 9]. AgNPs have been used for a wide range of healthcare products such as cosmetics, cleansing compounds, and medical devices/agents [10, 11]. However, our understanding of the underlying mechanisms of the antibacterial effects of AgNPs is still in its infantry. The killing action of AgNPs has been proposed to be the interaction of Ag^+^ with components on the bacterial membrane, leading to membrane, DNA, and protein damage [12, 13]. Moreover, AgNPs can induce high levels of reactive oxygen species (ROS) in the intracellular compartment of bacteria, causing damage to organic compounds within the cell [14].

Melioidosis is a severe tropical disease that is endemic in Southeast Asia and Northern Australia and potentially endemic in other tropical areas of the globe, but underreported [15, 16]. A recent study has estimated that the number of melioidosis cases worldwide may have been as high as 165,000 cases, with 89,000 deaths [17]. Melioidosis has various clinical manifestations ranging from localized skin infection to acute pneumonia and whole body sepsis. Relapse is common in melioidosis patients. Its etiological agent, *Burkholderia pseudomallei*, is a Gram-negative soil bacterium that can live freely in soil and water. *B*. *pseudomallei* is resistant to most antibiotics used in the empirical management of sepsis, complicating the antibacterial therapy for melioidosis. Although several studies have reported that various antimicrobial peptides can be used to treat *B*. *pseudomallei*, success has generally been obtained only *in vitro* [18, 19]. There is no vaccine for melioidosis, and it can be fatal if a specific antibiotic regimen is not delivered [20]. The standard antibiotics for the treatment of melioidosis are third-generation cephalosporins, including ceftazidime (CAZ). *B*. *pseudomallei* is intrinsically resistant to many antibiotics, and prolonged melioidosis treatment increases the probability of the bacteria acquiring further drug resistance [21], especially when mono therapy is used or the same type of antibiotic is used repeatedly in the same patient for the treatment of relapses. The emerging resistance of some *B*. *pseudomallei* strains to CAZ has recently been reported [22]. This serious problem, along with the paucity of alternate treatment options for melioidosis, has encouraged researchers to seek novel candidate agents to overcome *B*. *pseudomallei* [23, 24].

To the best of our knowledge, nanoparticles have not been used as alternative agents for treating *B*. *pseudomallei*. Considering that *B*. *pseudomallei* is not only a pathogen of neglected tropical diseases but also a potential biological weapon with intrinsic drug resistance, the development of a powerful treatment for this disease is urgently needed. The goal of this research is to find a novel antimicrobial agent against *B*. *pseudomallei*. We reported here the antimicrobial activity and mode of action of AgNPs against *B*. *pseudomallei*.

## Materials and Methods

### Preparation of silver nanoparticles

Spherical silver nanoparticles of 10–20 nm in size were obtained from a manufacturer (Prime Nanotechnology, Bangkok, Thailand) in the form of a 1 mg/mL suspension in deionized water. The AgNPs were characterized by spectroscopy and transmission electron microscopy. The plasmon extinction spectra of the AgNPs were recorded by an Ocean Optics USB4000 Fiber Optic Spectrometer coupled with a DH-2000 (Bangkok, Thailand) deuterium/halogen light source. All samples were diluted with deionized water to achieve a final AgNP concentration of 0.1 mg/mL. Transmission electron micrographs of the AgNPs were taken by a transmission electron microscope (TEM) (Hitachi Model H-7650) operating at 100 kV. To clean the AgNPs purchased from the manufacturer, the suspension was first centrifuged and then re-suspended in deionized water. The cleaned AgNPs were drop-casted onto a formvar-coated copper grid (200 mesh) and dried at room temperature in a desiccator for 24 hrs before TEM measurement. The dimensions of the silver nanoparticles were measured directly from the TEM micrographs using Image J software, a Java program developed by the National Institute of Mental Health [25].

### Bacterial strains and growth conditions

Nine strains of *Burkholderia pseudomallei* were provided by the Melioidosis Research Center, Khon Kaen University. Details of these strains are shown in Table 1. All strains were stored at -70°C in 20% glycerol in microcentrifuge tubes until use. The bacteria were streaked on nutrient agar (NA) and then cultured at 37°C overnight. Colonies were selected and inoculated in 5 mL of Luria-Bertani (LB) broth (no salt) at 37°C in an incubator overnight and then subcultured in 50 mL of the same medium at 37°C in a 190 rpm shaker-incubator for 2–3 h to yield a mid-logarithmic growth phase culture, which were used to investigate the antibacterial activity and mechanism of action [26].

**Table 1:** Strains of *B*. *pseudomallei* used in this study.

Strains	Sources	References
1026b	Clinical isolate from blood	[59]
K96243	Clinical isolate from blood	[60]
NF10/38	Clinical isolate from blood	[61]
EPMK31	Clinical isolate from plasma	[62]
H777	Clinical isolate from blood	[26]
316c	Clinical isolate from blood	[19]
979b	Clinical isolate from blood	[19]
EPMN34	Clinical isolate from plasma	[62]
EPMN159	Clinical isolate from blood	[62]

### Minimum inhibitory concentration (MIC) and minimum bactericidal concentration (MBC) assays

The MIC and MBC were determined by a serial dilution method in a 96-well plate using LB medium (Sigma–Aldrich) at an inoculum of 1x10^7^ CFU/mL of the tested bacteria. Nine strains of *B*. *pseudomallei* were treated with 2–256 μg/mL of AgNPs or 2–1,024 μg/mL of CAZ, and the antibacterial activity was determined 24 h later. Each treated condition was brought to 50 μL for a serial ten-fold dilution plate count (10^−1^–10^−8^ conc.) with sterile double-distilled deionized water in triplicate. Then, 10 μL of each dilution was spread on LB agar and cultured at 37°C overnight to count the bacterial colonies formed. Sterile double-distilled water was used as a no-treatment control. The MIC value corresponds to the lowest concentration that inhibits 99% of bacterial growth, and the MBC value corresponds to the lowest concentration that inhibits 100% of the bacterial growth. The percent inhibition was calculated using the formula [1-(CFU sample/CFU control)]×100 [27].

### Kinetic assay for antibacterial activity

Kinetic changes in the antibacterial activity of AgNPs and CAZ were determined by a serial dilution method in microplates using Luria–Bertani medium (Sigma–Aldrich) at an inoculum of 1x10^7^ CFU/mL. For this purpose, *B*. *pseudomallei* NF10 and 316c strains were re-suspended in 10 mM potassium phosphate buffer. The bacterial suspension was treated with AgNPs or CAZ at the final MIC and MBC. The bacterial cells were incubated at 37°C with shaking at 180 rpm. At 0, 1, 2, 4, 6 and 24 h of incubation, bacterial enumeration by plate counts were performed in triplicate. The bactericidal effect was defined as a ≥3 log10 reduction in CFU/mL compared with the initial inoculum. The kinetic changes within the first one hr were determined using a fluorescence microscope (Nikon, Eclipse). To visualize cell death, the LIVE/DEAD BacLight^TM^ bacterial viability kit (Invitrogen^®^) was used. This kit consists of two-color fluorescence probes, Sytox® Green for viable cells and propidium iodide (PI) for dead cells, allowing the simultaneous determination of live and dead cells. In brief, *B*. *pseudomallei* NF10 and 316c suspensions in 10 mM PPB (1x10^5^ CFU/mL) were incubated with AgNPs at MIC in a 180 rpm shaker-incubator at 37°C. The cell suspension was sampled at 0, 5, 30 and 60 min of incubation, stained with fluorescent dye using the LIVE/DEAD kit for 15 min in the dark, and smeared on glass slides. Fluorescence images were taken under a fluorescence microscope using excitation and emission wavelengths of 494 and 515 nm for Sytox® Green and 528 and 617 nm for propidium iodide, respectively [28].

### Hemolytic activity

To prepare a type O human red blood cell (hRBC) suspension, 20 mL of venous blood from a healthy volunteer was collected, washed three times with PBS, and then resuspended to 4% (v/v) in PBS. Aliquots of 100 μL hRBC suspensions were added into each well of a sterile 96-well plate. Then, 100 μL of AgNPs or CAZ at 2–1,024 μg/mL was added. The plates were incubated at 37°C for 1 h prior to centrifugation at 1,000 g for 5 min. 100 μL of the supernatant from each well was transferred to fresh 96-well plates, and the amount of released hemoglobin was measured as the absorbance at 405 nm using a SpectraMax M5 fluorescence microplate reader. The absorbance in the wells of the hRBCs incubated with PBS and 0.1% Triton X-100 were used as negative and positive controls, respectively. The percent hemolysis of RBCs was calculated according to the equation: percent hemolysis = ((sample absorbance−negative control absorbance)/(positive control absorbance−negative control absorbance))× 100. Less than 10% hemolysis were represented as non-toxic effect level in our experiments [29].

### Surface Charge Measurements

Zeta potential studies were performed at room temperature using a Zetasizer Nano ZS, as previously described [30]. Dilutions of the silver nanoparticles were prepared to final concentrations of 8, 32, 64, 96, 128 and 256 μg/mL with DI water. A volume of 100 μL of each AgNP stock dilution was added to 900 μL of the *B*. *pseudomallei* cells. Filtered sterile buffer was used as a positive control. The bacterial suspensions were added to disposable zeta cells with gold electrodes and allowed to equilibrate for 1 hr at 25°C. The zeta potential was measured and calculated per the manual instructions. The experiment was carried out twice for each AgNP concentration with independently grown cultures.

### Silver ion release measurement

Silver ion-releasing kinetics of AgNPs was performed by inductively coupled plasma optical emission spectrometry (ICP-OES)[1, 2], using Luria–Bertani medium (Sigma–Aldrich) at an inoculum of 1x10^7^ CFU/mL. *B*. *pseudomallei* 316c were re-suspended with deionized water. The bacterial suspension was treated with AgNPs and AgNO_3_ at MIC (48 μg/mL). The bacterial cells were incubated at 37°C with shaking at 180 rpm. At 0, 0.5, 1, 4 and 24 h of incubation, cell suspension was collected and released Ag^+^ was filtrated by Amicon® Ultra-30K concentrator (pore size of membrane as 7 nm) at 4,000 rpm centrifugation for 15 min. Flow-through fraction was quantified with ICP-OES (Agilent 7500c, Central Laboratory, Khon Kaen, Thailand) for Ag^+^ release.

### ROS production assay

The reactive oxygen species (ROS) induction by the AgNPs in *B*. *pseudomallei* strains NF10 and 316C and *E*. *coli* O157:H7 (a reference strain) were measured using an oxidation-sensitive fluorescent probe, 2′,7′-Dichlorodihydrofluorescein diacetate (DCFH-DA). DCFH-DA passively diffuses through the cell membrane into the cells and is deacetylated by esterases to form non-fluorescent 2′,7′-Dichlorodihydrofluorescein (DCFH). The DCFH reacts with ROS to form the fluorescent product 2′,7′-Dichlorofluorescin (DCF) trapped inside the cell, making the cell fluorescent. For this experiment, the bacteria cells were collected by centrifugation at 10,000 g for 15 min, diluted to 10^7^ CFU/mL, and then washed three times with 10 mM PPB. The DCFH-DA was mixed with the cell suspension at a ratio of 1:200, and the mixture was shaken for 60 min at 37°C to allow the fluorescent probe to get into the cells. After loading the fluorescent probe, the cell suspension was pelleted by centrifugation and washed twice to remove the extracellular probes. The cell suspension was exposed to different concentrations of AgNPs, as described in the previous section. The fluorescence intensity of the DCF was detected by a fluorescence spectrophotometer (SpectraMax M5) at excitation and emission wavelengths of 488 and 535 nm, respectively [14].

### Ultrastructural changes

The effects of AgNPs at low and high concentrations (1/5 MIC and MBC) on the morphology of the *B*. *pseudomallei* were examined under transmission electron microscopy (TEM), and the characterization of the AgNPs was determined by energy dispersive X-ray analysis (EDX). EDX was used to confirm the elemental presence of AgNPs in the electron micrographs. In brief, cells of *B*. *pseudomallei* 316c before and after treatment with AgNPs were fixed overnight with 2.5% glutaraldehyde. Samples were post-fixed in 2% osmium tetroxide, dehydrated in an ascending series of graded ethanol, infiltrated and embedded in spur resin. Then, ultra-thin sections (60 nm thicknesses) were cut by an ultramicrotome (Leica, EM UC7), stained with uranyl acetate and counter-stained with 4% lead citrate. These sections were mounted on carbon-coated copper grids and observed under TEM (Hitachi HT7700) [31].

### Statistical analysis

Pearson correlation coefficients by one-way ANOVA (significant: P < 0.05) were applied for statistical analysis (SPSS program version 17; Inc. Thailand). Data are presented as mean ± SDs in triplicated by origin Pro8 program.

## Results

### Characterization of the silver nanoparticles

**Fig 1:**
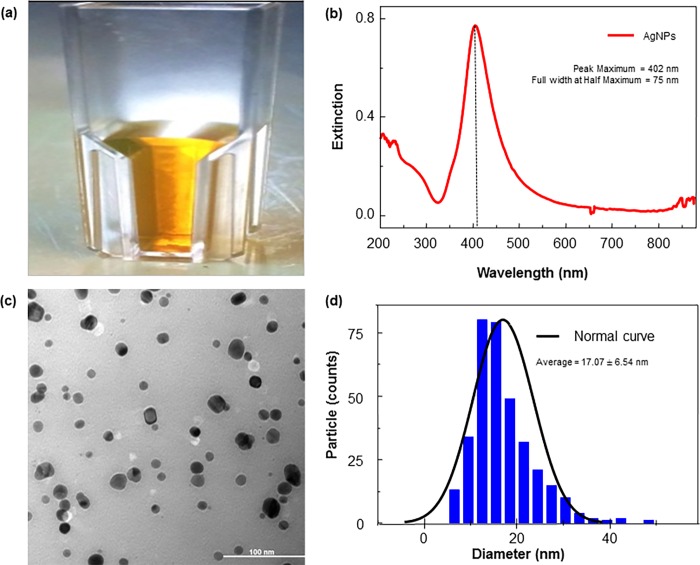
Characterization of AgNPs produced by chemical synthesis. (a) AgNP solution, (b) UV–vis absorption spectrum of AgNPs (b), TEM image of spherical AgNPs (c), particle size distributions by Image J software (d).

A monodispersion of AgNPs after synthesis is desirable to obtain the maximum anti-bacterial effect. In this study, the AgNPs were diluted from 10 to 1 mg/mL for physical characterization. The AgNP colloids appeared as a dark yellow color suspension without visible aggregation (Fig 1A). The spectrum scan of the AgNP colloidal solution yielded a single strong peak with an absorption maximum at approximately 400–410 nm, indicating the presence of AgNPs (Fig 1B). The TEM micrograph showed that the AgNPs had a spherical shape with an average size of 10–20 nm (Fig 1C and 1D).

### Minimum inhibitory concentration (MIC) and minimum bactericidal concentration (MBC) of AgNPs

Nine *B*. *pseudomallei* strains were tested for their MIC and MBC against AgNPs *in vitro*. Their MIC and MBC values were found to be 32–48 μg/mL and 96–128 μg/mL, respectively. The MIC and MBC values of CAZ are higher than those of the AgNPs, 128–512 μg/mL and 512–1024 μg/mL, respectively (Table 2).

**Table 2:** Minimum inhibitory concentration (MIC) and minimum bactericidal concentration (MBC) values of AgNPs and CAZ against *B*. *pseudomallei* in nine clinical isolates by serial dilution plate count assay.

Strain	AgNPs (μg/mL)		CAZ (μg/mL)	
	MIC	MBC	MIC	MBC
* 1026b	48	96	128	512
* K9624	48	96	128	512
* NF10	48	128	128	512
* EPMK31	48	96	128	512
** H777	48	128	256	1,024
*** 316c	32	96	512	1,024
*** 979b	32	96	512	1,024
*** EPMN34	48	128	512	1,024
*** EPMN159	48	96	512	1,024

The MIC value corresponds to the concentration that inhibited >99% of bacterial growth, and the MBC value corresponds to the concentration that inhibited 100% of the bacterial growth.

*(CAZ non-resistant isolates)

**(CAZ moderately resistant isolates)

***(CAZ highly resistant isolates)

### Kinetics of bactericidal activity of AgNPs versus CAZ

**Fig 2:**
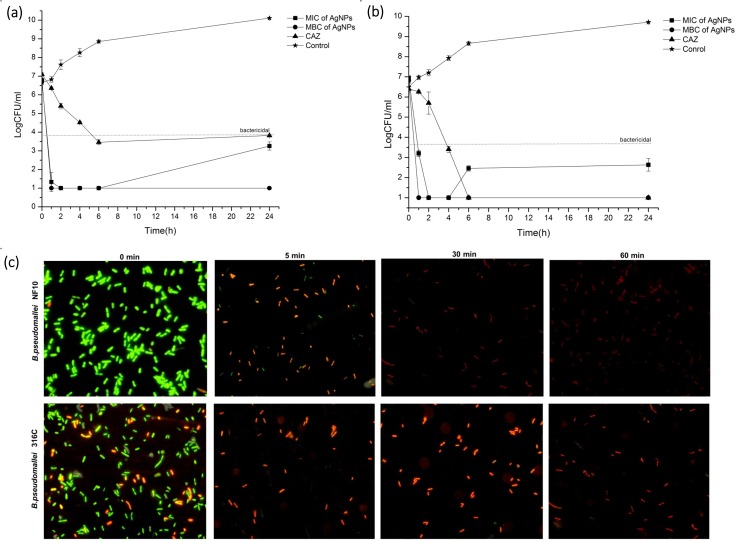
Killing kinetics of AgNPs against *B*. *pseudomallei*. Bacterial suspension strains NF10 (a) or 316a (b) were incubated with various concentrations of AgNPs and CAZ for 1, 2, 4, 6 and 24 h. Colonies were counted, and the bactericidal effects were defined as a ≥3-log reduction in colony-forming units (CFU)/mL compared to the initial inoculum. Short-term effects of AgNPs against *B*. *pseudomallei* NF10 and 316c were determined 0, 5, 30 and 60 min after exposure to AgNPs using a LIVE/DEAD^®^ BacLightTM bacteria viability kit by fluorescence microscopy (c). Data represent the means ± standard errors of the means for triplicate samples. Results shown are representative of two independent experiments.

We examined the kinetics of the bactericidal activity of AgNPs in comparison with that of CAZ on two selected *B*. *pseudomallei* strains, NF10 and 316c, the CAZ-sensitive and resistant strains, respectively. The study revealed that AgNPs at the MIC and MBC exerted a rapid bactericidal activity of ≥3-log within 1 hr of exposure against both *B*. *pseudomallei* strains, whereas CAZ required up to 6 hrs (Fig 2A and 2B). To investigate early killing event, the effect of the AgNPs on the bacterial viability within the first hr was investigated using fluorescence microscopy with the LIVE/DEAD^®^ kit. As shown in Fig 2C, the AgNPs showed a rapid killing effect against *B*. *pseudomallei* within the first 5 min, which can be recognized as the green fluorescence in viable cells at 0 min turning to the red color of dead cells within 5 min. The smaller size of the dead cells when treated with AgNPs demonstrated morphological changes and cell shrinkage. At the later times of treatment (30 and 60 min), the red cells disappeared, indicating cell lysis.

### Hemolytic activity of AgNPs

**Fig 3:**
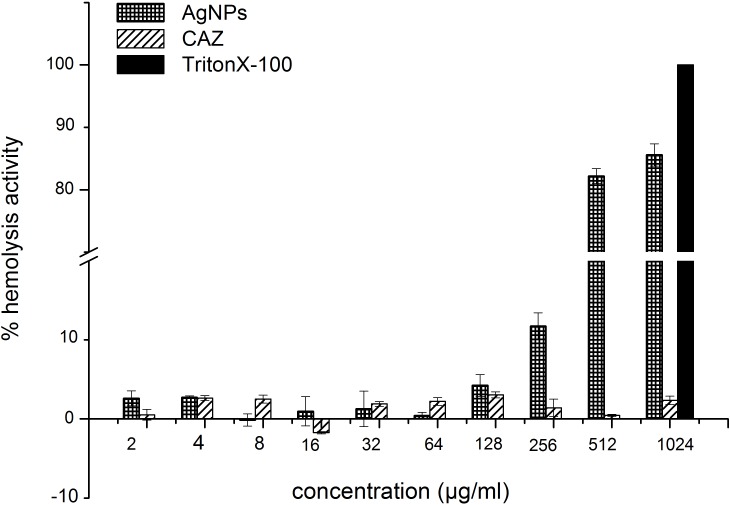
Hemolytic activity of AgNPs. Human erythrocytes were incubated in PBS with various concentrations of AgNPs for 1 hr at 37°C. The hemoglobin release was monitored using a microplate reader at the absorbance at 405 nm. Data represent the means ± standard errors of the means for triplicate samples. Results shown are representative of two independent experiments.

To investigate the potential clinical applicability of AgNPs, their cytotoxicity against human cells was assessed by hemolysis using human RBCs. As shown in Fig 3, the AgNPs did not cause hemolysis in the concentration range of the MIC and MBC (<10%) against *B*. *pseudomallei*, although it caused hemolysis of more than 10% of the cells at a higher concentration (256 μg/mL).

### Surface charge neutralization of *B*. *pseudomalle*i by AgNPs

**Fig 4:**
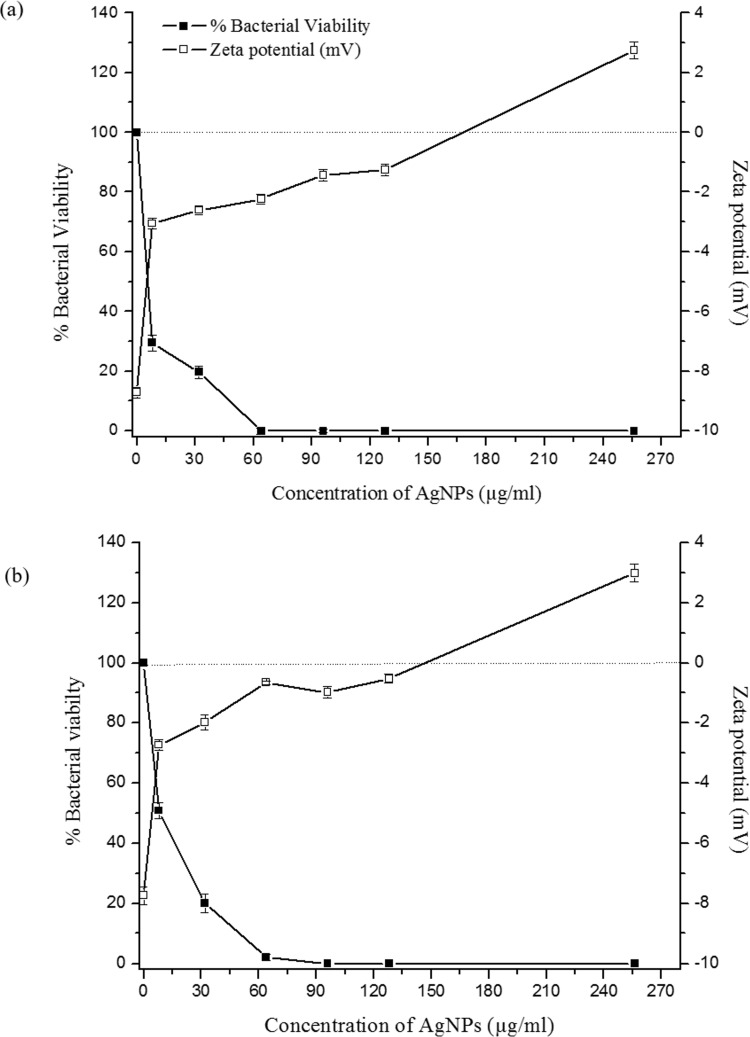
Effect of AgNP on bacterial viability and cell-surface charge of *B*. *pseudomallei*. *B*. *pseudomallei* NF10 (a) and 316c (b) were treated with AgNP concentrations of 8, 32, 64, 96, 128 and 256 μg/mL. Black squares corresponds to the percentage of viable bacterial cells in the presence of increasing AgNPs concentrations, whereas the zeta potential is indicated by the white squares. The dotted line indicates a neutral surface net charge, to highlight the AgNP concentration range at which both strains of *B*. *pseudomallei* exhibit surface neutrality and possible overcompensation is achieved. Data represent the means ± standard errors of the means for triplicate samples. Results shown are representative of two independent experiments.

The result in Fig 4A and 4B shows that under normal conditions present differences in the zeta potential between the bacterial surface and the medium, on the membrane surface was -8.93 and -7.95 mV for *B*. *pseudomallei* NF10 and 316c, respectively. When treated with AgNPs at concentrations of 8, 32, 64, 96, 128 and 256 μg/mL, the resultant zeta potentials were similar in both strains and could be observed as 3 phases. First, at 8 μg/mL AgNP treatment, the cell surfaces showed a fast rate of neutralization, i.e., an increase of the zeta potential toward zero. Then, the neutralization steadily increased at higher concentration AgNP treatments, and at 128–256 μg/mL, the rate of neutralization rapidly rose again. Overall, the zeta potential values increased from -8.73 to 2.85 and -7.74 to 2.94 mV for *B*. *pseudomallei* NF10 and 316c, respectively. The drop in bacterial viability corresponded well with the increase of the zeta potential, which reached zero at 128–256 μg/mL AgNPs.

### Silver ion release of AgNPs after *B*. *pseudomallei* exposure

**Fig 5:**
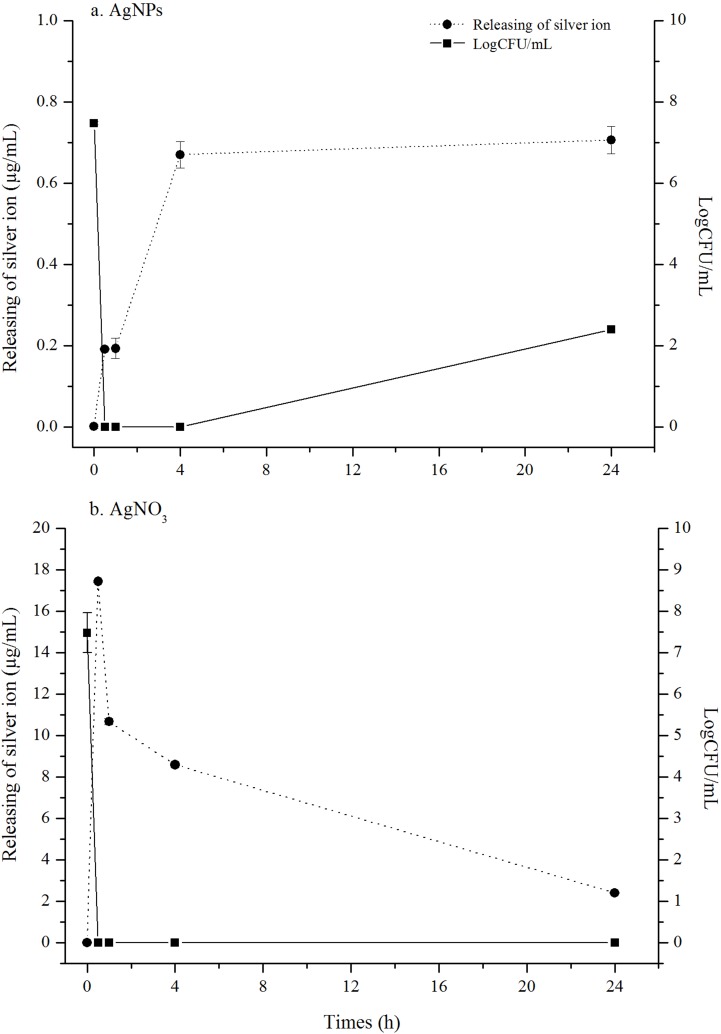
Kinetics of silver ion release on *B*.*pseudomallei* 316c. *B*.*pseudomallei* 316c was treated with AgNPs and AgNO_3_ at 48 μg/mL for 0, 0.5,1,4 and 24 h at 37°C. The silver ion release was measured using ICP-OES technique. The killing kinetic was measured using dilution plate count method. Both of data are the mean of two experiments performed in triplicate.

Ag^+^ release after exposing AgNPs to *B*. *pseudomallei* suspension was followed as a function of time using ICP-OES measurement (Fig 5A). In first 4 h, Ag^+^ concentration rapidly increased from 0.0013 to 0.67 μg/mL, with the starting concentration of AgNPs at 48 μg/mL at 0 h. The release was slowing down and reached 0.71 μg/mL at 24 h. When correlating cell growth inhibition with Ag^+^ release, the growth was rapidly dropped within 30 min, coinciding with Ag^+^ release at 0.19 μg/mL. In the control experiment, Ag^+^ released from AgNO_3_ resulted in a complete drop of cell growth at ion concentration of 17.43 μg/mL.

### ROS induction by AgNPs

**Fig 6:**
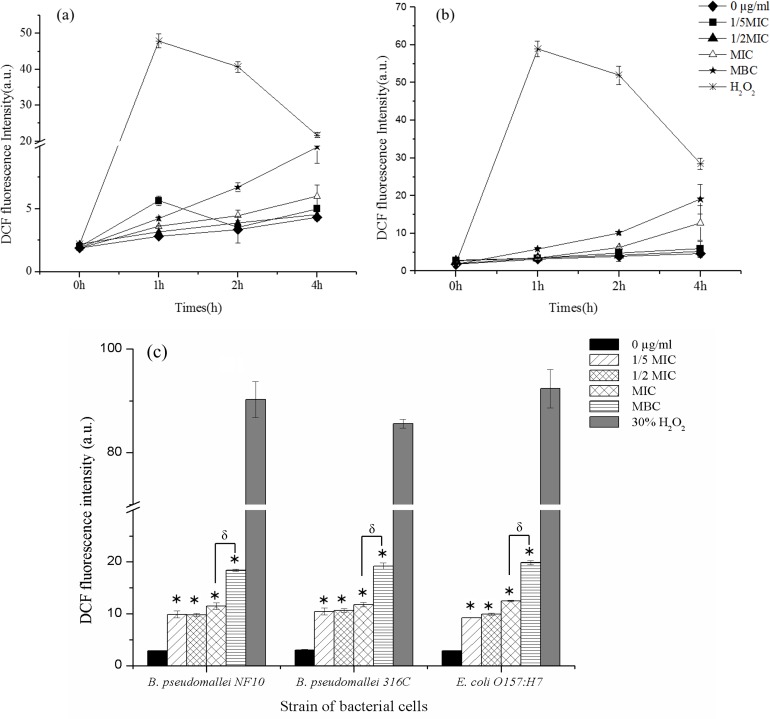
Kinetics of ROS induction in *B*. *pseudomallei* after exposure to AgNPs. Kinetics of induction of ROS in *B*. *pseudomallei* NF10 (a) and 316c (b) and accumulated ROS at 4 hrs (c) were followed by DCF fluorescence. All bacterial cells were treated with various concentrations of AgNPs: 0 μg/mL, 1/5 MIC, 1/2 MIC, MIC and MBC of AgNPs. 30% H_2_O_2_ was used as a positive control. The overall P-value was determined using one-way ANOVA. * indicates a P value of <0.001 in comparison to bacterial cells treated with deionize water (0 μg/mL), δ indicates a P value of < 0.007 in comparison of MIC with MBC treated condition. Data represent the means standard errors of the means for triplicate samples. Results shown are representative of two independent experiments.

Because AgNPs are known to induce the accumulation of reactive oxygen species (ROS) inside bacterial cells, the ROS production in two strains of *B*. *pseudomallei*, NF10 and 316c, and *E*. *coli* O157:H7 (reference bacterial cell) after exposure to AgNPs was measured using the DCFH-DA method. ROS were produced slowly within the first two hrs after the AgNP treatment, with a marked increase in ROS found 3–4 hrs after treatment, with the MBC generally enhancing the ROS production (Fig 6A and 6B). At concentrations of 1/5 MIC, 1/2 MIC and MIC, the AgNPs induced ROS concentrations approximately 3 times higher than the control (p values < 0.001). When treated with AgNPs at the MBC (Fig 6C), ROS concentration increased approximately 5 times higher than control (p values < 0.001). The ROS production appeared to be strain-independent, like the MIC and killing kinetics results.

### AgNP-induced ultrastructural changes

**Fig 7:**
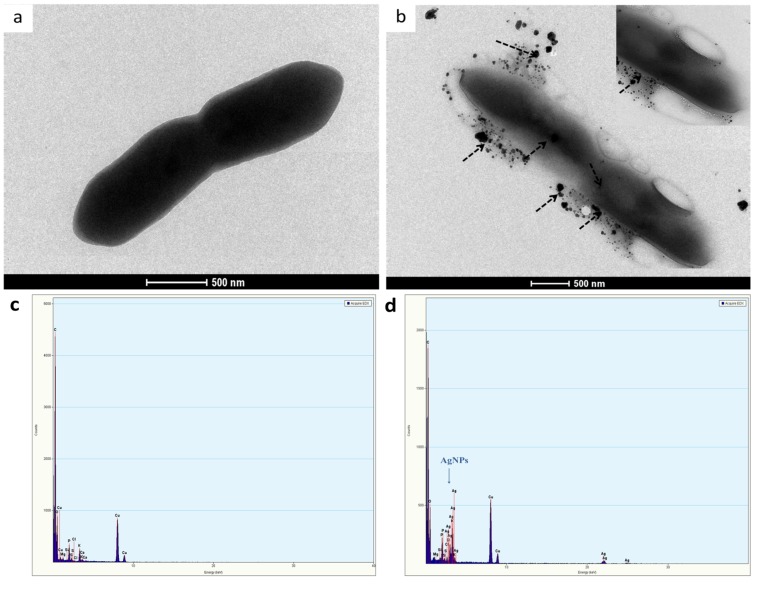
TEM micrographs and EDX spectra of *B*. *pseudomallei* 316c. (a) normal cell in 10 mM PPB; (b) bacterial cell treated with 1/5 MIC of AgNPs for 1 h at 37°C (dashed arrows point to the AgNPs on the outer membrane and inside the cell); (c) EDX spectra of the untreated cells and (d) EDX spectra of the cells treated with 1/5 MIC of AgNPs.

The effects of AgNPs on the bacterial cell membrane were examined using TEM. As shown in Fig 7A, the control *B*. *pseudomallei* culture showed a normal conformation and smooth membrane structure. When bacterial cells were treated with 1/5 of the AgNP MIC, the AgNPs accumulated and dispersed on the outer surface of the cells (Fig 7B). The EDX spectra in Fig 6D identify the particles on the membrane of the bacterial cells as AgNPs.

**Fig 8:**
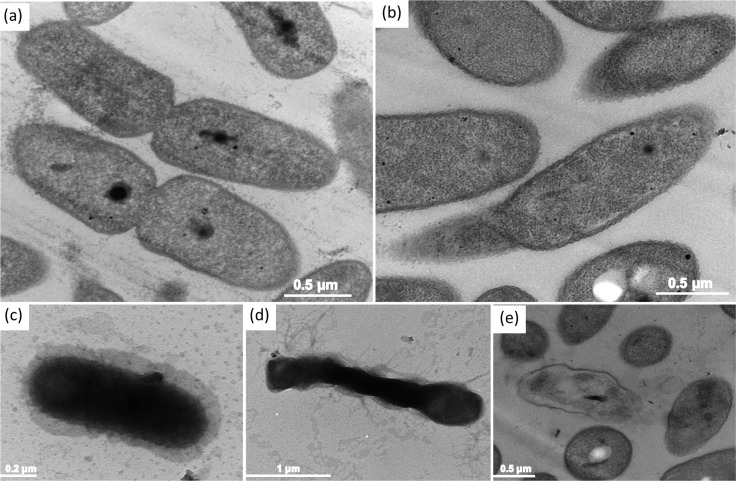
Ultrastructural changes of *B*. *pseudomallei* 316c cells after exposure to AgNPs. TEM micrographs prepared by ultramicrotome for ultra-thin sections show (a) untreated cells, (b-d) cells exposed to 1/5 MIC of AgNPs and (e) cells exposed to MBC for 1 hr at 37°C. Dashed arrows show nucleoid of *B*. *pseudomallei* 316c, and asterisk indicates bacterial vacuole.

In addition, we observed the ultrastructural response of the bacterial cells after treatment with AgNPs through an ultrathin section using an ultramicrotome. The TEM micrograph of an ultra-thin section of a normal cell (Fig 8A) shows a rod shape, smooth outer membrane, and clumped DNA. The electron density was uniform in normal bacterial cells.

When the cells were exposed to 1/5 MIC of AgNPs for 1 hr, the TEM micrographs showed that damage to the cell membrane led to the leakage of cytoplasm. As observed in Fig 7B–7D the cell presented an amorphous shape and an empty or ghost structure. In some cells, an electron-lucent space (asterisk in Fig 8C and 8D) was presented in the cytoplasm, change to suggesting the leakage of various biomolecules outside of the cytoplasm. When treated at higher concentrations of AgNPs (MBC) in Fig 8E, the bacterial cells disappeared, leaving visible cellular debris.

## Discussion

As several pathogenic bacteria are developing antibiotic resistance, AgNPs are considered a new material with the hope of treating them. AgNPs are used for targeted drug delivery to combat antibiotic-resistant pathogens and increase the efficacy of treatment [2, 13, 27]. There has been a tremendous increase in the application of silver and AgNPs such as in home consumer products, clothing and fabrics, food instruments, disinfectants and medical devices [32, 33]. Evidence of the AgNP antibacterial activity and mechanism of action against antibiotic-resistant pathogens has been reported [5, 34, 35].

In this study, we observed the antimicrobial activity of AgNPs against nine strains of *B*. *pseudomallei*, including CAZ-sensitive and CAZ-resistant phenotypes. AgNPs had a higher antibacterial activity than CAZ (Table 1). Because there are no previous reports on the use of AgNPs against *B*. *pseudomallei*, we can compare only the MIC and MBC values with the reported results of pathogenic bacteria such as *Pseudomonas aeruginosa* that share an ancestry with *B*. *pseudomallei*. We found that the MIC and MBC values of AgNPs against all strains of *B*. *pseudomallei* in this study were higher than those observed on *P*. *aeruginosa* in the past. For example, the antibacterial effect of AgNPs on multi drug resistant *P*. *aeruginosa* was reported have MIC_50_ and MIC values of 15 and 50 μg/mL, respectively [36]. In another study, the MIC and MBC values of AgNPs against drug-resistant bacteria were reported to be 79.4 μM and 83.3 μM, respectively [27]. AgNPs of approximately 10–20 nm in size showed MIC values of approximately 6–28 μg/mL against Gram-negative bacteria [37]. The higher MICs found in our study probably emerged from the existence of silver resistance genes, active efflux and the outer membrane composition of *B*. *pseudomallei* [38–40].

We took interest in following the kinetics of killing after treating *B*. *pseudomallei* with AgNPs. The time-kill assay indicated that cell death transpired within 30 min after the AgNP treatment (Fig 2A and 2B). However, as visualized under fluorescence microscopy, almost all the cells were dead within 5 min of AgNP treatment in both strains (Fig 2C). We demonstrated here evidence of the fast killing action of AgNPs. Our killing kinetics also showed us that the number of dead cells after treatment with AgNPs was much higher than for those treated with CAZ. This finding indicates the advantage of using AgNPs to reduce the risk of recurrence of *B*. *pseudomallei* (Fig 2A and 2B). Nevertheless, an in vivo study is required to support our findings.

We followed changes of the cell surface charge, as an increase in the surface charge is a strong indicator of a drop in cell viability (Fig 4). The outer membrane of *B*. *pseudomallei* has distinct characteristics that are not present in that of *E*. *coli*. One such characteristic is the modification of the biphosphorylated disaccharide backbone of lipid A, one of the structural moieties of lipopolysaccharides (LPS), with 4-amino-4-deoxy-arabinose (Ara4N). These modifications reduce the net negative charge of LPS, making the cell zeta potential closer to neutral, since each phosphate group contributes a -1 charge at neutral pH [41–43]. Only the phospholipid head groups on the outer membrane contribute for the surface charge of *B*. *pseudomallei*. The overall cell surface charge of *B*. *pseudomallei* is therefore not as negative as *E*. *coli*. According to our results, the zeta potential of both *B*. *pseudomallei* strains, regardless of CAZ susceptibility, was found to be around -8 mV. In comparison, other studies have determined the zeta potential of *E*. *coli* under normal conditions to be ~-20 mV, which was similar to ours [44].

The reduction of the zeta potential of *B*. *pseudomallei* towards 0 mV indicates charge neutralization in the presence of AgNPs. The neutralization is potentially due to the electrostatic binding of positively charged Ag+ with the negatively charged phospholipids in the outer membrane [45]. This leads to the cell membrane being depolarized, resulting in uncontrollable ion transport across the membrane. It appears from our evidence that the cell death coincides with the surface charge neutralization. The neutralization effect found in our study is similar to the study of Alves et.al. (2010) when the bacterial cells were treated with cationic antimicrobial peptides (CAMPs) [44].

The effect of AgNPs against bacteria, and in this study representing *B*. *pseudomallei*, is mediated via multiple mechanisms [46, 47]. From the vast number of collective studies on the antibacterial mechanism of AgNPs, three mechanisms of bacterial cell killing are widely discussed. 1) AgNPs deposit on the bacterial surface, interfering with the permeability of the cell membrane, 2) AgNPs penetrate into the cytosolic space, causing damage to key macromolecules including DNA and proteins, 3) AgNPs dissolution in the presence of oxygen leads to oxidation reactions which allow the release of Ag+. The reactive Ag+ can subsequently interact with sulphur-containing proteins, resulting in loss of function. If the target proteins are involved in key metabolic pathways of the cell, ATP production may be compromised. Ag+ is also a key precursor in inducing reactive oxygen species (ROS), which are believed to be one of the major causes of cellular damage [48–51]. To current knowledge, all these proposed mechanisms suggest the combined antibacterial activity of AgNPs and its oxidized species, Ag+. It was found that Ag+ alone (AgNO_3_) had lower antibacterial activity than the same equivalent concentration of AgNPs [52, 53]. High local concentrations of released Ag+ from AgNPs maybe the contributing factor for the observed higher activity than the more evenly distributed Ag+ from AgNO_3_ in the solution.

Our experimental evidence of Ag^+^ release from AgNPs is another main highlighted finding besides the two-phase mechanism of AgNPs in this study. The results in Fig 5A, to our surprise, indicates that the Ag^+^ release was present in a very small fraction when comparing to the starting concentration of AgNPs at 48 μg/mL. At 30 min where the cell growth was completely inhibited, the Ag^+^ was found to be only 0.19 μg/mL, i.e. 3.9% of total Ag element in the cell suspension. AgNPs, in contrast, was present at about 96% in the cell suspension. Although reported in previous studies that both AgNPs and Ag^+^ take action together in bactericidal activity, this study adds another layer of insights by demonstrating quantitatively the amount of Ag+ released from AgNPs after interacting with the bacterial cells and that AgNPs mainly contributing in the killing action.

AgNPs are reported to have an effect on the membrane morphology [54, 55]. In this study, at the MIC value of AgNPs, the membrane and biomolecules were found to be severely damaged within 5–30 min, as indicated by PI staining (Fig 2C). As shown in Fig 7, the TEM technique can be used to detect nanoparticle dissolution, agglomeration, and re-precipitation. TEM micrographs showed different ultra-structural responses for sub-MIC and MBC values of the AgNPs (Fig 8). In the sub-MIC range, we found conformational changes such as “articulate cell” due to the abnormal turgor pressure of the cells, leading to the formation of membrane debris, cytoplasm release and cell death, as reported previously [10, 13, 31, 56]. In contrast, at the MBC, we speculated that the apoptosis-like response in *B*. *pseudomallei* is caused by the generation of ROS (Fig 8E). Bacterial cells produce ROS intracellular metabolites under normal conditions. When the bacterial cells are situated under stress conditions, they will produce many ROS molecules such as superoxide anions (O_2_^-^), hydrogen peroxide (H_2_O_2_) and hydroxyl radicals (OH^-^). All these steps are believed to have an effect on the several steps of the apoptosis cascade [57]. ROS can also induce a bacterial apoptosis-like response by causing damage to cellular components [58].

In addition to using TEM images as indirect evidence of ROS-induced cell destruction, we measured the direct ROS production by following the increase of DCF fluorescence at different concentrations of AgNPs in a time-dependent manner (Fig 6). Surprisingly, ROS were produced long after cell death. Although cell death was observed within 5–30 min, ROS were slowly produced within the first hr. Prominent production occurred 4 hr after the AgNP treatment. The levels of ROS using AgNPs at sub-MIC and MIC were similar (Fig 6C), whereas it was much higher at the MBC, which indicates a positive correlation between the AgNP concentration and ROS production that persisted even some time after cell death.

### Model summarizing mechanism of silver nanoparticles (AgNPs) against *B*. *pseudomallei*

**Fig 9:**
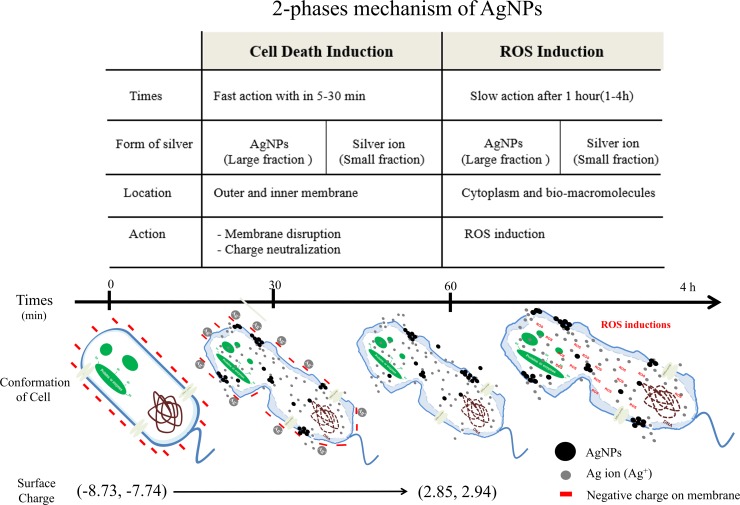
Proposed two-phase mechanism of AgNPs against *B*. *pseudomallei*. The two-phase mechanism consists of a cell death induction phase and ROS induction phase. In the cell death induction phase, AgNPs induced rapid cell death within 5–30 minutes via the action of large fraction of AgNPs and small fraction of Ag^+^. The total charge of the membrane was altered from -8.73 and-7.74 to 2.85 and 2.94 mV due to the neutralization of the positively charged Ag^+^ by negatively charged molecules on the surface of the bacterial cells. Outer and inner membrane disruption was evidenced by the fluorescence probe PI. The leakage of cytoplasm and damage of bio-macromolecules was evidenced by a TEM micrograph taken at one hr after AgNP treatment. In the ROS induction phase, a slow response of ROS production was found after 1 hr.

Based on the present results, we postulate a two-phase mechanism of AgNPs against *B*. *pseudomallei*, as illustrated in Fig 9. The first phase involves cell death induction, where the cells were very abruptly killed by AgNPs within 5–30 min (Fig 2). During the killing mechanism, the AgNPs release a small fraction of Ag^+^. Both forms can bind with electrostatic force to the negatively charged phospholipids of the outer membrane, leading to surface charge neutralization and also cell membrane disruption (Fig 4, Fig 8). Consequently, most of the AgNPs can penetrate and agglomerate in the cytoplasm of the cells (Fig 7). We found cell leakage and membrane damage in the TEM micrographs and the PI staining (Fig 8, Fig 8C), all within a 1 h timeframe. The second phase takes place after the first phase, with ROS induction found 1–4 h after the AgNP treatment. These ROS were slowly accumulated within the first hr (Fig 6A and 6B) and then prominently increased within 4 hr. The slow accumulation of ROS in the first hr is probably due to a very small fraction of Ag^+^ release after AgNPs making a contact with and/or penetrate through the bacterial cells (Fig 5A). A prominent ROS found at 4 hr is the accumulating products of the continuing oxidative chain reaction, albeit the very low concentration of Ag^+^ at the beginning. In addition, the ROS accumulation in the second phase implies the secondary action of the Ag^+^ after their primary action in the initial killing phase.

## Conclusions

In summary, we conclude that AgNPs (10–20 nm) have better antimicrobial activity than CAZ in both CAZ-sensitive and CAZ-resistant *B*. *pseudomallei*. AgNPs are not toxic to mammalian cells at bactericidal concentrations. The mechanism of action of AgNPs involves 2 phases: cell death induction and ROS induction. Both AgNPs and Ag^+^ contribute to the killing effect, with the much larger proportion of AgNPs present. This research provides an initial study of *B*. *pseudomallei* inhibition using AgNPs and elucidates the mechanism involved. The timeline mapping of the AgNP action proposed here could provide essential information for the designing of a melioidosis treatment regimen using AgNPs in combination with a conventional protocol.

## Supporting Information

S1 Data UV-Vis intensity of AgNPs. (DOCX)

S2 Data Raw data of Fig 2–Fig 6. (DOCX)

S3 Data SPSS data of Fig 6C. (RTF)
