# Supplementary material for: Two-Phase Bactericidal Mechanism of Silver Nanoparticles against Burkholderia pseudomallei
Source: PLoS One. 2016 Dec 15;11(12):e0168098. doi: 10.1371/journal.pone.0168098 (PMC5158019; doi:10.1371/journal.pone.0168098)
Supplement: S1 Data — (DOCX) [file pone.0168098.s001.docx]

**Raw data of Fig 1b**

SpectraSuite Data File

++++++++++++++++++++++++++++++++++++

Date: Tue Aug 26 16:35:16 ICT 2014

User: SRU

Dark Spectrum Present: Yes

Reference Spectrum Present: Yes

Number of Sampled Component Spectra: 1

Spectrometers: USB2G09354

Integration Time (usec): 5000 (USB2G09354)

Spectra Averaged: 128 (USB2G09354)

Boxcar Smoothing: 10 (USB2G09354)

Correct for Electrical Dark: No (USB2G09354)

Strobe/Lamp Enabled: No (USB2G09354)

Correct for Detector Non-linearity: No (USB2G09354)

Correct for Stray Light: No (USB2G09354)

Number of Pixels in Processed Spectrum: 2048

>>>>>Begin Processed Spectral Data<<<<<

179.55 -0.000

179.93 -0.000

180.30 -0.202

180.68 0.054

181.05 -0.753

181.43 -0.014

181.80 -0.097

182.18 -0.014

182.55 0.064

182.92 0.026

183.30 -0.018

183.67 -0.013

184.05 0.013

184.42 0.002

184.80 0.034

185.17 0.072

185.54 0.082

185.92 0.093

186.29 0.090

186.67 0.101

187.04 0.106

187.42 0.108

187.79 0.111

188.16 0.101

188.54 0.106

188.91 0.114

189.29 0.114

189.66 0.114

190.03 0.115

190.41 0.115

190.78 0.118

191.15 0.119

191.53 0.121

191.90 0.120

192.28 0.124

192.65 0.124

193.02 0.123

193.40 0.127

193.77 0.125

194.14 0.126

194.52 0.124

194.89 0.123

195.27 0.119

195.64 0.116

196.01 0.121

196.39 0.122

196.76 0.119

197.13 0.121

197.51 0.120

197.88 0.119

198.25 0.121

198.63 0.124

199.00 0.126

199.37 0.127

199.75 0.126

200.12 0.123

200.49 0.123

200.87 0.123

201.24 0.119

201.61 0.123

201.99 0.126

202.36 0.128

202.73 0.130

203.11 0.130

203.48 0.134

203.85 0.134

204.23 0.138

204.60 0.139

204.97 0.138

205.34 0.138

205.72 0.135

206.09 0.136

206.46 0.131

206.84 0.129

207.21 0.127

207.58 0.128

207.95 0.129

208.33 0.129

208.70 0.131

209.07 0.136

209.45 0.135

209.82 0.135

210.19 0.133

210.56 0.131

210.94 0.132

211.31 0.134

211.68 0.132

212.05 0.130

212.43 0.130

212.80 0.128

213.17 0.127

213.54 0.129

213.92 0.126

214.29 0.127

214.66 0.125

215.03 0.126

215.41 0.125

215.78 0.126

216.15 0.126

216.52 0.127

216.90 0.125

217.27 0.127

217.64 0.124

218.01 0.126

218.38 0.127

218.76 0.130

219.13 0.125

219.50 0.127

219.87 0.124

220.24 0.125

220.62 0.129

220.99 0.131

221.36 0.130

221.73 0.130

222.10 0.129

222.48 0.129

222.85 0.131

223.22 0.128

223.59 0.127

223.96 0.124

224.34 0.121

224.71 0.122

225.08 0.119

225.45 0.121

225.82 0.120

226.19 0.120

226.57 0.121

226.94 0.125

227.31 0.123

227.68 0.125

228.05 0.125

228.42 0.124

228.79 0.125

229.17 0.127

229.54 0.127

229.91 0.131

230.28 0.133

230.65 0.134

231.02 0.140

231.39 0.141

231.77 0.144

232.14 0.146

232.51 0.148

232.88 0.149

233.25 0.150

233.62 0.151

233.99 0.152

234.36 0.151

234.74 0.153

235.11 0.155

235.48 0.155

235.85 0.156

236.22 0.157

236.59 0.158

236.96 0.159

237.33 0.161

237.70 0.162

238.07 0.161

238.44 0.161

238.82 0.161

239.19 0.162

239.56 0.163

239.93 0.165

240.30 0.165

240.67 0.166

241.04 0.166

241.41 0.167

241.78 0.168

242.15 0.169

242.52 0.169

242.89 0.169

243.26 0.169

243.63 0.170

244.00 0.170

244.37 0.170

244.75 0.170

245.12 0.171

245.49 0.170

245.86 0.171

246.23 0.171

246.60 0.171

246.97 0.171

247.34 0.171

247.71 0.170

248.08 0.170

248.45 0.170

248.82 0.170

249.19 0.170

249.56 0.170

249.93 0.170

250.30 0.170

250.67 0.169

251.04 0.169

251.41 0.169

251.78 0.169

252.15 0.169

252.52 0.169

252.89 0.168

253.26 0.168

253.63 0.168

254.00 0.168

254.37 0.167

254.74 0.167

255.11 0.167

255.48 0.167

255.85 0.167

256.22 0.166

256.59 0.166

256.95 0.166

257.32 0.165

257.69 0.165

258.06 0.165

258.43 0.165

258.80 0.164

259.17 0.164

259.54 0.164

259.91 0.164

260.28 0.163

260.65 0.163

261.02 0.163

261.39 0.163

261.76 0.162

262.13 0.162

262.50 0.162

262.86 0.161

263.23 0.161

263.60 0.160

263.97 0.160

264.34 0.160

264.71 0.159

265.08 0.159

265.45 0.158

265.82 0.158

266.19 0.157

266.56 0.157

266.92 0.156

267.29 0.156

267.66 0.155

268.03 0.155

268.40 0.154

268.77 0.154

269.14 0.153

269.51 0.153

269.87 0.152

270.24 0.151

270.61 0.151

270.98 0.150

271.35 0.150

271.72 0.149

272.09 0.149

272.45 0.148

272.82 0.148

273.19 0.147

273.56 0.146

273.93 0.146

274.30 0.145

274.66 0.144

275.03 0.144

275.40 0.143

275.77 0.142

276.14 0.142

276.51 0.141

276.87 0.141

277.24 0.140

277.61 0.140

277.98 0.139

278.35 0.138

278.71 0.138

279.08 0.137

279.45 0.136

279.82 0.136

280.19 0.135

280.55 0.135

280.92 0.134

281.29 0.133

281.66 0.133

282.03 0.132

282.39 0.131

282.76 0.131

283.13 0.130

283.50 0.130

283.86 0.129

284.23 0.128

284.60 0.128

284.97 0.127

285.34 0.126

285.70 0.126

286.07 0.125

286.44 0.124

286.81 0.124

287.17 0.123

287.54 0.122

287.91 0.122

288.27 0.121

288.64 0.120

289.01 0.119

289.38 0.119

289.74 0.118

290.11 0.117

290.48 0.116

290.85 0.115

291.21 0.115

291.58 0.114

291.95 0.113

292.31 0.112

292.68 0.111

293.05 0.110

293.41 0.109

293.78 0.109

294.15 0.108

294.52 0.107

294.88 0.106

295.25 0.105

295.62 0.104

295.98 0.103

296.35 0.102

296.72 0.101

297.08 0.099

297.45 0.098

297.82 0.097

298.18 0.096

298.55 0.095

298.92 0.094

299.28 0.093

299.65 0.092

300.02 0.091

300.38 0.089

300.75 0.088

301.12 0.087

301.48 0.086

301.85 0.084

302.21 0.083

302.58 0.082

302.95 0.081

303.31 0.080

303.68 0.079

304.05 0.078

304.41 0.077

304.78 0.075

305.14 0.074

305.51 0.073

305.88 0.072

306.24 0.071

306.61 0.070

306.97 0.069

307.34 0.067

307.71 0.066

308.07 0.065

308.44 0.064

308.80 0.063

309.17 0.062

309.53 0.061

309.90 0.060

310.27 0.059

310.63 0.058

311.00 0.057

311.36 0.056

311.73 0.056

312.09 0.055

312.46 0.054

312.83 0.053

313.19 0.052

313.56 0.051

313.92 0.051

314.29 0.050

314.65 0.049

315.02 0.049

315.38 0.048

315.75 0.047

316.11 0.047

316.48 0.046

316.84 0.046

317.21 0.045

317.57 0.045

317.94 0.044

318.30 0.044

318.67 0.044

319.03 0.043

319.40 0.043

319.76 0.043

320.13 0.042

320.49 0.042

320.86 0.042

321.22 0.042

321.59 0.042

321.95 0.042

322.32 0.041

322.68 0.041

323.05 0.041

323.41 0.041

323.78 0.041

324.14 0.041

324.51 0.041

324.87 0.041

325.24 0.042

325.60 0.042

325.97 0.042

326.33 0.042

326.69 0.042

327.06 0.043

327.42 0.043

327.79 0.044

328.15 0.044

328.52 0.045

328.88 0.045

329.24 0.046

329.61 0.046

329.97 0.047

330.34 0.048

330.70 0.048

331.07 0.049

331.43 0.050

331.79 0.051

332.16 0.052

332.52 0.052

332.89 0.053

333.25 0.054

333.61 0.055

333.98 0.057

334.34 0.058

334.70 0.059

335.07 0.060

335.43 0.061

335.80 0.063

336.16 0.064

336.52 0.065

336.89 0.067

337.25 0.068

337.61 0.070

337.98 0.071

338.34 0.073

338.70 0.075

339.07 0.077

339.43 0.078

339.79 0.080

340.16 0.082

340.52 0.084

340.88 0.086

341.25 0.087

341.61 0.089

341.97 0.091

342.34 0.093

342.70 0.095

343.06 0.097

343.43 0.099

343.79 0.101

344.15 0.103

344.52 0.105

344.88 0.108

345.24 0.110

345.61 0.112

345.97 0.114

346.33 0.116

346.69 0.118

347.06 0.120

347.42 0.122

347.78 0.124

348.15 0.126

348.51 0.128

348.87 0.129

349.23 0.131

349.60 0.133

349.96 0.135

350.32 0.137

350.68 0.139

351.05 0.140

351.41 0.142

351.77 0.144

352.13 0.145

352.50 0.147

352.86 0.148

353.22 0.150

353.58 0.152

353.94 0.153

354.31 0.155

354.67 0.156

355.03 0.158

355.39 0.159

355.76 0.161

356.12 0.163

356.48 0.164

356.84 0.166

357.20 0.167

357.57 0.169

357.93 0.170

358.29 0.172

358.65 0.173

359.01 0.175

359.37 0.177

359.74 0.179

360.10 0.180

360.46 0.182

360.82 0.184

361.18 0.186

361.55 0.188

361.91 0.190

362.27 0.192

362.63 0.194

362.99 0.196

363.35 0.199

363.71 0.201

364.08 0.203

364.44 0.206

364.80 0.208

365.16 0.211

365.52 0.213

365.88 0.216

366.24 0.219

366.60 0.221

366.97 0.224

367.33 0.227

367.69 0.230

368.05 0.233

368.41 0.236

368.77 0.239

369.13 0.242

369.49 0.245

369.85 0.248

370.22 0.251

370.58 0.254

370.94 0.258

371.30 0.261

371.66 0.265

372.02 0.269

372.38 0.272

372.74 0.276

373.10 0.280

373.46 0.284

373.82 0.288

374.18 0.292

374.54 0.296

374.90 0.300

375.26 0.304

375.63 0.309

375.99 0.313

376.35 0.317

376.71 0.322

377.07 0.326

377.43 0.330

377.79 0.335

378.15 0.339

378.51 0.344

378.87 0.348

379.23 0.353

379.59 0.358

379.95 0.363

380.31 0.368

380.67 0.372

381.03 0.377

381.39 0.382

381.75 0.387

382.11 0.392

382.47 0.397

382.83 0.403

383.19 0.408

383.55 0.413

383.91 0.419

384.27 0.424

384.63 0.430

384.99 0.436

385.35 0.441

385.70 0.447

386.06 0.452

386.42 0.457

386.78 0.462

387.14 0.467

387.50 0.473

387.86 0.478

388.22 0.483

388.58 0.488

388.94 0.494

389.30 0.499

389.66 0.505

390.02 0.510

390.38 0.515

390.74 0.520

391.09 0.525

391.45 0.529

391.81 0.534

392.17 0.539

392.53 0.543

392.89 0.548

393.25 0.552

393.61 0.556

393.97 0.560

394.33 0.564

394.68 0.568

395.04 0.572

395.40 0.576

395.76 0.580

396.12 0.583

396.48 0.586

396.84 0.589

397.19 0.592

397.55 0.595

397.91 0.597

398.27 0.600

398.63 0.602

398.99 0.604

399.35 0.606

399.70 0.608

400.06 0.609

400.42 0.611

400.78 0.612

401.14 0.614

401.50 0.615

401.85 0.616

402.21 0.617

402.57 0.617

402.93 0.618

403.29 0.618

403.64 0.618

404.00 0.618

404.36 0.618

404.72 0.618

405.08 0.617

405.43 0.617

405.79 0.616

406.15 0.616

406.51 0.615

406.86 0.614

407.22 0.613

407.58 0.612

407.94 0.611

408.29 0.610

408.65 0.608

409.01 0.606

409.37 0.604

409.72 0.602

410.08 0.600

410.44 0.598

410.80 0.596

411.15 0.594

411.51 0.592

411.87 0.589

412.23 0.587

412.58 0.585

412.94 0.582

413.30 0.579

413.65 0.577

414.01 0.574

414.37 0.571

414.73 0.569

415.08 0.566

415.44 0.563

415.80 0.560

416.15 0.557

416.51 0.555

416.87 0.552

417.22 0.548

417.58 0.545

417.94 0.542

418.29 0.539

418.65 0.536

419.01 0.532

419.36 0.530

419.72 0.527

420.08 0.523

420.43 0.520

420.79 0.517

421.15 0.514

421.50 0.511

421.86 0.509

422.22 0.506

422.57 0.503

422.93 0.500

423.28 0.497

423.64 0.494

424.00 0.491

424.35 0.488

424.71 0.485

425.06 0.482

425.42 0.478

425.78 0.475

426.13 0.472

426.49 0.468

426.84 0.464

427.20 0.461

427.56 0.458

427.91 0.454

428.27 0.451

428.62 0.448

428.98 0.446

429.33 0.443

429.69 0.440

430.05 0.437

430.40 0.434

430.76 0.431

431.11 0.428

431.47 0.425

431.82 0.422

432.18 0.419

432.53 0.417

432.89 0.414

433.24 0.411

433.60 0.408

433.95 0.405

434.31 0.401

434.67 0.398

435.02 0.395

435.38 0.392

435.73 0.389

436.09 0.386

436.44 0.383

436.80 0.380

437.15 0.378

437.51 0.375

437.86 0.372

438.22 0.369

438.57 0.366

438.92 0.363

439.28 0.361

439.63 0.358

439.99 0.355

440.34 0.353

440.70 0.350

441.05 0.348

441.41 0.345

441.76 0.343

442.12 0.341

442.47 0.338

442.82 0.336

443.18 0.333

443.53 0.331

443.89 0.329

444.24 0.327

444.60 0.325

444.95 0.323

445.30 0.320

445.66 0.318

446.01 0.315

446.37 0.313

446.72 0.311

447.07 0.309

447.43 0.306

447.78 0.304

448.14 0.302

448.49 0.300

448.84 0.297

449.20 0.295

449.55 0.293

449.91 0.291

450.26 0.289

450.61 0.287

450.97 0.285

451.32 0.282

451.67 0.280

452.03 0.278

452.38 0.276

452.73 0.274

453.09 0.272

453.44 0.270

453.79 0.268

454.15 0.266

454.50 0.264

454.85 0.262

455.21 0.260

455.56 0.258

455.91 0.256

456.27 0.254

456.62 0.252

456.97 0.250

457.33 0.249

457.68 0.247

458.03 0.245

458.38 0.244

458.74 0.242

459.09 0.240

459.44 0.239

459.80 0.237

460.15 0.235

460.50 0.234

460.85 0.232

461.21 0.230

461.56 0.229

461.91 0.227

462.26 0.226

462.62 0.224

462.97 0.222

463.32 0.221

463.67 0.219

464.03 0.218

464.38 0.216

464.73 0.215

465.08 0.213

465.43 0.212

465.79 0.211

466.14 0.209

466.49 0.208

466.84 0.207

467.20 0.206

467.55 0.204

467.90 0.203

468.25 0.202

468.60 0.200

468.95 0.199

469.31 0.197

469.66 0.195

470.01 0.194

470.36 0.192

470.71 0.191

471.07 0.189

471.42 0.188

471.77 0.186

472.12 0.185

472.47 0.184

472.82 0.182

473.17 0.181

473.53 0.180

473.88 0.179

474.23 0.178

474.58 0.177

474.93 0.176

475.28 0.175

475.63 0.174

475.98 0.173

476.34 0.172

476.69 0.171

477.04 0.170

477.39 0.169

477.74 0.168

478.09 0.167

478.44 0.166

478.79 0.165

479.14 0.164

479.49 0.163

479.84 0.162

480.20 0.161

480.55 0.160

480.90 0.159

481.25 0.158

481.60 0.157

481.95 0.155

482.30 0.154

482.65 0.152

483.00 0.151

483.35 0.150

483.70 0.150

484.05 0.149

484.40 0.148

484.75 0.148

485.10 0.147

485.45 0.147

485.80 0.146

486.15 0.146

486.50 0.145

486.85 0.145

487.20 0.144

487.55 0.144

487.90 0.143

488.25 0.143

488.60 0.142

488.95 0.141

489.30 0.141

489.65 0.140

490.00 0.139

490.35 0.138

490.70 0.137

491.05 0.136

491.40 0.135

491.75 0.134

492.10 0.133

492.45 0.132

492.80 0.131

493.15 0.130

493.50 0.130

493.85 0.129

494.20 0.128

494.54 0.127

494.89 0.126

495.24 0.126

495.59 0.125

495.94 0.124

496.29 0.124

496.64 0.123

496.99 0.122

497.34 0.121

497.69 0.121

498.04 0.120

498.38 0.120

498.73 0.119

499.08 0.118

499.43 0.118

499.78 0.117

500.13 0.116

500.48 0.116

500.83 0.115

501.17 0.115

501.52 0.114

501.87 0.114

502.22 0.113

502.57 0.112

502.92 0.112

503.26 0.111

503.61 0.111

503.96 0.110

504.31 0.109

504.66 0.109

505.01 0.108

505.35 0.108

505.70 0.107

506.05 0.107

506.40 0.106

506.75 0.105

507.09 0.105

507.44 0.104

507.79 0.104

508.14 0.103

508.49 0.102

508.83 0.102

509.18 0.101

509.53 0.101

509.88 0.100

510.22 0.100

510.57 0.099

510.92 0.099

511.27 0.098

511.61 0.098

511.96 0.097

512.31 0.097

512.66 0.096

513.00 0.096

513.35 0.095

513.70 0.095

514.05 0.095

514.39 0.094

514.74 0.093

515.09 0.093

515.43 0.092

515.78 0.092

516.13 0.091

516.48 0.091

516.82 0.090

517.17 0.090

517.52 0.090

517.86 0.089

518.21 0.089

518.56 0.088

518.90 0.088

519.25 0.087

519.60 0.087

519.94 0.086

520.29 0.086

520.64 0.085

520.98 0.085

521.33 0.084

521.68 0.084

522.02 0.084

522.37 0.083

522.71 0.083

523.06 0.082

523.41 0.082

523.75 0.081

524.10 0.081

524.45 0.081

524.79 0.080

525.14 0.080

525.48 0.079

525.83 0.079

526.18 0.079

526.52 0.078

526.87 0.078

527.21 0.078

527.56 0.077

527.90 0.077

528.25 0.077

528.60 0.077

528.94 0.076

529.29 0.076

529.63 0.076

529.98 0.075

530.32 0.075

530.67 0.075

531.01 0.074

531.36 0.074

531.70 0.074

532.05 0.073

532.40 0.073

532.74 0.073

533.09 0.073

533.43 0.072

533.78 0.072

534.12 0.072

534.47 0.072

534.81 0.071

535.16 0.071

535.50 0.071

535.85 0.071

536.19 0.070

536.54 0.070

536.88 0.070

537.22 0.070

537.57 0.070

537.91 0.069

538.26 0.069

538.60 0.069

538.95 0.069

539.29 0.068

539.64 0.068

539.98 0.068

540.33 0.068

540.67 0.067

541.01 0.067

541.36 0.067

541.70 0.067

542.05 0.066

542.39 0.066

542.74 0.066

543.08 0.066

543.42 0.065

543.77 0.065

544.11 0.065

544.46 0.064

544.80 0.064

545.14 0.064

545.49 0.064

545.83 0.064

546.17 0.063

546.52 0.063

546.86 0.063

547.21 0.062

547.55 0.062

547.89 0.062

548.24 0.062

548.58 0.061

548.92 0.061

549.27 0.061

549.61 0.061

549.95 0.060

550.30 0.060

550.64 0.060

550.98 0.060

551.33 0.060

551.67 0.060

552.01 0.059

552.35 0.059

552.70 0.059

553.04 0.058

553.38 0.058

553.73 0.058

554.07 0.058

554.41 0.058

554.75 0.057

555.10 0.057

555.44 0.057

555.78 0.057

556.13 0.057

556.47 0.056

556.81 0.056

557.15 0.056

557.50 0.056

557.84 0.055

558.18 0.055

558.52 0.055

558.86 0.055

559.21 0.055

559.55 0.054

559.89 0.054

560.23 0.054

560.58 0.054

560.92 0.054

561.26 0.054

561.60 0.053

561.94 0.053

562.29 0.053

562.63 0.053

562.97 0.053

563.31 0.053

563.65 0.052

563.99 0.052

564.34 0.052

564.68 0.052

565.02 0.052

565.36 0.051

565.70 0.051

566.04 0.051

566.39 0.051

566.73 0.050

567.07 0.050

567.41 0.050

567.75 0.049

568.09 0.049

568.43 0.049

568.77 0.049

569.12 0.049

569.46 0.048

569.80 0.048

570.14 0.048

570.48 0.048

570.82 0.048

571.16 0.048

571.50 0.047

571.84 0.047

572.18 0.047

572.52 0.047

572.86 0.047

573.21 0.047

573.55 0.047

573.89 0.046

574.23 0.046

574.57 0.046

574.91 0.046

575.25 0.046

575.59 0.046

575.93 0.045

576.27 0.045

576.61 0.045

576.95 0.045

577.29 0.045

577.63 0.045

577.97 0.044

578.31 0.044

578.65 0.044

578.99 0.044

579.33 0.044

579.67 0.043

580.01 0.043

580.35 0.043

580.69 0.043

581.03 0.043

581.37 0.043

581.71 0.043

582.05 0.043

582.39 0.043

582.73 0.042

583.07 0.042

583.41 0.042

583.75 0.042

584.09 0.042

584.42 0.042

584.76 0.042

585.10 0.042

585.44 0.042

585.78 0.042

586.12 0.041

586.46 0.041

586.80 0.041

587.14 0.041

587.48 0.041

587.82 0.041

588.15 0.041

588.49 0.041

588.83 0.041

589.17 0.041

589.51 0.040

589.85 0.040

590.19 0.040

590.53 0.040

590.86 0.040

591.20 0.040

591.54 0.040

591.88 0.040

592.22 0.039

592.56 0.039

592.89 0.039

593.23 0.039

593.57 0.039

593.91 0.039

594.25 0.039

594.59 0.039

594.92 0.039

595.26 0.039

595.60 0.039

595.94 0.039

596.28 0.039

596.61 0.038

596.95 0.038

597.29 0.038

597.63 0.038

597.96 0.038

598.30 0.038

598.64 0.038

598.98 0.038

599.31 0.038

599.65 0.037

599.99 0.037

600.33 0.037

600.66 0.037

601.00 0.037

601.34 0.036

601.68 0.036

602.01 0.036

602.35 0.036

602.69 0.036

603.03 0.036

603.36 0.036

603.70 0.036

604.04 0.036

604.37 0.036

604.71 0.035

605.05 0.035

605.38 0.035

605.72 0.035

606.06 0.035

606.39 0.035

606.73 0.035

607.07 0.035

607.40 0.035

607.74 0.035

608.08 0.035

608.41 0.035

608.75 0.035

609.09 0.035

609.42 0.034

609.76 0.034

610.09 0.034

610.43 0.034

610.77 0.034

611.10 0.034

611.44 0.034

611.78 0.034

612.11 0.034

612.45 0.034

612.78 0.034

613.12 0.034

613.45 0.034

613.79 0.034

614.13 0.033

614.46 0.033

614.80 0.033

615.13 0.033

615.47 0.033

615.80 0.033

616.14 0.033

616.48 0.033

616.81 0.033

617.15 0.033

617.48 0.033

617.82 0.033

618.15 0.032

618.49 0.032

618.82 0.032

619.16 0.032

619.49 0.032

619.83 0.032

620.16 0.032

620.50 0.032

620.83 0.032

621.17 0.031

621.50 0.031

621.84 0.031

622.17 0.032

622.51 0.031

622.84 0.031

623.18 0.031

623.51 0.031

623.84 0.031

624.18 0.031

624.51 0.031

624.85 0.031

625.18 0.031

625.52 0.031

625.85 0.031

626.18 0.031

626.52 0.031

626.85 0.030

627.19 0.030

627.52 0.030

627.86 0.030

628.19 0.030

628.52 0.030

628.86 0.030

629.19 0.030

629.52 0.030

629.86 0.030

630.19 0.030

630.53 0.030

630.86 0.029

631.19 0.029

631.53 0.029

631.86 0.029

632.19 0.029

632.53 0.029

632.86 0.029

633.19 0.029

633.53 0.029

633.86 0.029

634.19 0.029

634.53 0.029

634.86 0.028

635.19 0.028

635.53 0.028

635.86 0.029

636.19 0.029

636.52 0.028

636.86 0.028

637.19 0.029

637.52 0.028

637.86 0.028

638.19 0.028

638.52 0.028

638.85 0.028

639.19 0.028

639.52 0.028

639.85 0.028

640.18 0.028

640.52 0.028

640.85 0.028

641.18 0.028

641.51 0.028

641.85 0.028

642.18 0.028

642.51 0.027

642.84 0.027

643.17 0.027

643.51 0.027

643.84 0.027

644.17 0.027

644.50 0.027

644.83 0.027

645.17 0.027

645.50 0.027

645.83 0.027

646.16 0.027

646.49 0.027

646.82 0.027

647.16 0.026

647.49 0.027

647.82 0.026

648.15 0.026

648.48 0.026

648.81 0.027

649.14 0.027

649.48 0.027

649.81 0.027

650.14 0.027

650.47 0.026

650.80 0.026

651.13 0.026

651.46 0.026

651.79 0.026

652.12 0.027

652.45 0.017

652.79 0.012

653.12 0.009

653.45 0.007

653.78 0.005

654.11 0.005

654.44 0.004

654.77 0.009

655.10 0.010

655.43 0.011

655.76 0.011

656.09 0.012

656.42 0.012

656.75 0.012

657.08 0.012

657.41 0.012

657.74 0.012

658.07 0.012

658.40 0.012

658.73 0.012

659.06 0.012

659.39 0.012

659.72 0.014

660.05 0.016

660.38 0.019

660.71 0.022

661.04 0.027

661.37 0.036

661.70 0.026

662.03 0.026

662.36 0.026

662.69 0.026

663.02 0.025

663.35 0.025

663.68 0.025

664.01 0.025

664.34 0.024

664.67 0.024

664.99 0.024

665.32 0.024

665.65 0.024

665.98 0.024

666.31 0.024

666.64 0.024

666.97 0.024

667.30 0.024

667.63 0.024

667.95 0.024

668.28 0.024

668.61 0.024

668.94 0.024

669.27 0.024

669.60 0.023

669.93 0.023

670.26 0.024

670.58 0.024

670.91 0.024

671.24 0.024

671.57 0.024

671.90 0.023

672.23 0.024

672.55 0.023

672.88 0.024

673.21 0.023

673.54 0.023

673.87 0.023

674.19 0.023

674.52 0.023

674.85 0.023

675.18 0.023

675.51 0.023

675.83 0.023

676.16 0.023

676.49 0.023

676.82 0.023

677.14 0.023

677.47 0.023

677.80 0.023

678.13 0.022

678.45 0.022

678.78 0.022

679.11 0.022

679.43 0.022

679.76 0.022

680.09 0.022

680.42 0.022

680.74 0.022

681.07 0.022

681.40 0.022

681.72 0.022

682.05 0.022

682.38 0.022

682.70 0.022

683.03 0.022

683.36 0.022

683.68 0.022

684.01 0.022

684.34 0.022

684.66 0.022

684.99 0.022

685.32 0.022

685.64 0.022

685.97 0.022

686.30 0.022

686.62 0.022

686.95 0.021

687.28 0.021

687.60 0.021

687.93 0.021

688.25 0.021

688.58 0.021

688.91 0.021

689.23 0.021

689.56 0.021

689.88 0.020

690.21 0.020

690.53 0.020

690.86 0.020

691.19 0.020

691.51 0.020

691.84 0.020

692.16 0.021

692.49 0.020

692.81 0.020

693.14 0.020

693.46 0.020

693.79 0.021

694.11 0.021

694.44 0.021

694.76 0.021

695.09 0.021

695.41 0.021

695.74 0.021

696.06 0.021

696.39 0.020

696.71 0.021

697.04 0.021

697.36 0.021

697.69 0.021

698.01 0.020

698.34 0.020

698.66 0.020

698.99 0.020

699.31 0.020

699.64 0.020

699.96 0.020

700.28 0.020

700.61 0.020

700.93 0.020

701.26 0.020

701.58 0.020

701.91 0.020

702.23 0.020

702.55 0.020

702.88 0.020

703.20 0.021

703.53 0.021

703.85 0.021

704.17 0.021

704.50 0.021

704.82 0.021

705.14 0.021

705.47 0.021

705.79 0.021

706.12 0.021

706.44 0.021

706.76 0.021

707.09 0.021

707.41 0.021

707.73 0.021

708.06 0.021

708.38 0.021

708.70 0.021

709.02 0.021

709.35 0.021

709.67 0.021

709.99 0.021

710.32 0.021

710.64 0.020

710.96 0.020

711.29 0.020

711.61 0.020

711.93 0.020

712.25 0.020

712.58 0.020

712.90 0.020

713.22 0.020

713.54 0.020

713.87 0.020

714.19 0.020

714.51 0.019

714.83 0.020

715.16 0.020

715.48 0.019

715.80 0.020

716.12 0.019

716.44 0.019

716.77 0.019

717.09 0.019

717.41 0.019

717.73 0.019

718.05 0.019

718.38 0.019

718.70 0.019

719.02 0.019

719.34 0.019

719.66 0.019

719.98 0.020

720.30 0.019

720.63 0.019

720.95 0.019

721.27 0.019

721.59 0.019

721.91 0.019

722.23 0.019

722.55 0.019

722.87 0.019

723.20 0.019

723.52 0.019

723.84 0.019

724.16 0.019

724.48 0.019

724.80 0.019

725.12 0.019

725.44 0.019

725.76 0.018

726.08 0.018

726.40 0.019

726.72 0.018

727.05 0.019

727.37 0.018

727.69 0.018

728.01 0.018

728.33 0.018

728.65 0.018

728.97 0.018

729.29 0.018

729.61 0.018

729.93 0.018

730.25 0.018

730.57 0.017

730.89 0.017

731.21 0.017

731.53 0.017

731.85 0.017

732.17 0.017

732.49 0.017

732.81 0.017

733.13 0.016

733.45 0.016

733.76 0.016

734.08 0.016

734.40 0.016

734.72 0.016

735.04 0.016

735.36 0.016

735.68 0.016

736.00 0.016

736.32 0.016

736.64 0.016

736.96 0.016

737.28 0.016

737.60 0.016

737.91 0.016

738.23 0.016

738.55 0.016

738.87 0.016

739.19 0.016

739.51 0.016

739.83 0.016

740.15 0.016

740.46 0.016

740.78 0.016

741.10 0.016

741.42 0.016

741.74 0.016

742.06 0.016

742.37 0.016

742.69 0.016

743.01 0.016

743.33 0.016

743.65 0.016

743.96 0.016

744.28 0.016

744.60 0.016

744.92 0.015

745.24 0.015

745.55 0.015

745.87 0.015

746.19 0.015

746.51 0.015

746.82 0.015

747.14 0.015

747.46 0.015

747.78 0.015

748.09 0.015

748.41 0.015

748.73 0.015

749.05 0.015

749.36 0.015

749.68 0.015

750.00 0.015

750.31 0.015

750.63 0.015

750.95 0.015

751.26 0.016

751.58 0.016

751.90 0.016

752.22 0.016

752.53 0.016

752.85 0.016

753.17 0.016

753.48 0.016

753.80 0.016

754.11 0.016

754.43 0.015

754.75 0.015

755.06 0.015

755.38 0.015

755.70 0.015

756.01 0.015

756.33 0.015

756.64 0.015

756.96 0.015

757.28 0.015

757.59 0.015

757.91 0.015

758.22 0.015

758.54 0.015

758.86 0.015

759.17 0.015

759.49 0.015

759.80 0.015

760.12 0.015

760.43 0.015

760.75 0.015

761.06 0.015

761.38 0.015

761.70 0.015

762.01 0.015

762.33 0.015

762.64 0.015

762.96 0.015

763.27 0.015

763.59 0.015

763.90 0.015

764.22 0.015

764.53 0.015

764.85 0.015

765.16 0.015

765.47 0.015

765.79 0.015

766.10 0.015

766.42 0.015

766.73 0.015

767.05 0.015

767.36 0.015

767.68 0.015

767.99 0.015

768.30 0.014

768.62 0.014

768.93 0.014

769.25 0.014

769.56 0.014

769.88 0.014

770.19 0.014

770.50 0.014

770.82 0.014

771.13 0.014

771.44 0.014

771.76 0.014

772.07 0.014

772.39 0.014

772.70 0.013

773.01 0.013

773.33 0.013

773.64 0.013

773.95 0.013

774.27 0.013

774.58 0.013

774.89 0.013

775.21 0.013

775.52 0.013

775.83 0.013

776.15 0.013

776.46 0.013

776.77 0.013

777.08 0.013

777.40 0.013

777.71 0.013

778.02 0.013

778.34 0.013

778.65 0.012

778.96 0.012

779.27 0.012

779.59 0.012

779.90 0.012

780.21 0.013

780.52 0.013

780.83 0.013

781.15 0.012

781.46 0.012

781.77 0.012

782.08 0.012

782.40 0.012

782.71 0.012

783.02 0.013

783.33 0.013

783.64 0.013

783.96 0.013

784.27 0.013

784.58 0.013

784.89 0.013

785.20 0.013

785.51 0.012

785.82 0.012

786.14 0.013

786.45 0.013

786.76 0.012

787.07 0.013

787.38 0.013

787.69 0.013

788.00 0.013

788.31 0.013

788.63 0.013

788.94 0.013

789.25 0.012

789.56 0.012

789.87 0.012

790.18 0.012

790.49 0.012

790.80 0.012

791.11 0.012

791.42 0.012

791.73 0.012

792.04 0.012

792.35 0.012

792.67 0.012

792.98 0.012

793.29 0.012

793.60 0.012

793.91 0.012

794.22 0.012

794.53 0.012

794.84 0.012

795.15 0.012

795.46 0.012

795.77 0.012

796.08 0.012

796.39 0.012

796.70 0.012

797.01 0.012

797.31 0.013

797.62 0.012

797.93 0.012

798.24 0.012

798.55 0.012

798.86 0.012

799.17 0.012

799.48 0.013

799.79 0.013

800.10 0.013

800.41 0.013

800.72 0.013

801.03 0.012

801.34 0.012

801.64 0.012

801.95 0.012

802.26 0.012

802.57 0.012

802.88 0.012

803.19 0.013

803.50 0.013

803.81 0.012

804.11 0.012

804.42 0.012

804.73 0.012

805.04 0.012

805.35 0.012

805.66 0.012

805.96 0.012

806.27 0.012

806.58 0.012

806.89 0.012

807.20 0.012

807.50 0.012

807.81 0.012

808.12 0.011

808.43 0.012

808.74 0.012

809.04 0.012

809.35 0.012

809.66 0.011

809.97 0.011

810.27 0.011

810.58 0.011

810.89 0.011

811.20 0.011

811.50 0.011

811.81 0.011

812.12 0.012

812.42 0.011

812.73 0.011

813.04 0.010

813.35 0.010

813.65 0.010

813.96 0.010

814.27 0.010

814.57 0.011

814.88 0.011

815.19 0.011

815.49 0.011

815.80 0.011

816.11 0.011

816.41 0.011

816.72 0.011

817.03 0.011

817.33 0.011

817.64 0.011

817.94 0.011

818.25 0.011

818.56 0.011

818.86 0.011

819.17 0.011

819.47 0.011

819.78 0.011

820.09 0.011

820.39 0.011

820.70 0.011

821.00 0.010

821.31 0.010

821.61 0.010

821.92 0.010

822.23 0.010

822.53 0.010

822.84 0.010

823.14 0.010

823.45 0.010

823.75 0.009

824.06 0.009

824.36 0.009

824.67 0.010

824.97 0.009

825.28 0.009

825.58 0.009

825.89 0.009

826.19 0.009

826.50 0.010

826.80 0.010

827.11 0.010

827.41 0.010

827.72 0.009

828.02 0.009

828.32 0.009

828.63 0.009

828.93 0.009

829.24 0.009

829.54 0.010

829.85 0.010

830.15 0.011

830.45 0.010

830.76 0.009

831.06 0.009

831.37 0.009

831.67 0.011

831.97 0.011

832.28 0.011

832.58 0.011

832.89 0.010

833.19 0.009

833.49 0.010

833.80 0.011

834.10 0.011

834.40 0.010

834.71 0.009

835.01 0.010

835.31 0.010

835.62 0.010

835.92 0.010

836.22 0.010

836.53 0.010

836.83 0.011

837.13 0.012

837.43 0.012

837.74 0.011

838.04 0.010

838.34 0.009

838.65 0.010

838.95 0.009

839.25 0.009

839.55 0.008

839.86 0.008

840.16 0.007

840.46 0.008

840.76 0.008

841.06 0.007

841.37 0.006

841.67 0.005

841.97 0.006

842.27 0.007

842.57 0.006

842.88 0.007

843.18 0.006

843.48 0.005

843.78 0.005

844.08 0.006

844.39 0.004

844.69 0.005

844.99 0.006

845.29 0.006

845.59 0.007

845.89 0.008

846.19 0.009

846.50 0.010

846.80 0.009

847.10 0.012

847.40 0.013

847.70 0.014

848.00 0.014

848.30 0.013

848.60 0.011

848.90 0.013

849.21 0.011

849.51 0.013

849.81 0.011

850.11 0.010

850.41 0.009

850.71 0.010

851.01 0.010

851.31 0.010

851.61 0.010

851.91 0.009

852.21 0.010

852.51 0.010

852.81 0.010

853.11 0.010

853.41 0.010

853.71 0.010

854.01 0.010

854.31 0.011

854.61 0.011

854.91 0.011

855.21 0.011

855.51 0.010

855.81 0.010

856.11 0.010

856.41 0.010

856.71 0.012

857.01 0.012

857.31 0.012

857.61 0.012

857.91 0.011

858.20 0.011

858.50 0.010

858.80 0.010

859.10 0.009

859.40 0.009

859.70 0.008

860.00 0.009

860.30 0.008

860.60 0.008

860.90 0.007

861.19 0.006

861.49 0.005

861.79 0.005

862.09 0.005

862.39 0.006

862.69 0.006

862.98 0.005

863.28 0.004

863.58 0.004

863.88 0.005

864.18 0.005

864.48 0.007

864.77 0.009

865.07 0.010

865.37 0.011

865.67 0.011

865.97 0.010

866.26 0.009

866.56 0.009

866.86 0.008

867.16 0.008

867.45 0.008

867.75 0.011

868.05 0.011

868.35 0.012

868.64 0.012

868.94 0.013

869.24 0.013

869.54 0.013

869.83 0.013

870.13 0.013

870.43 0.014

870.72 0.010

871.02 0.011

871.32 0.010

871.61 0.008

871.91 0.009

872.21 0.010

872.50 0.010

872.80 0.009

873.10 0.011

873.39 0.011

873.69 0.010

873.99 0.011

874.28 0.019

874.58 -0.018

874.87 0.017

875.17 0.040

875.47 -0.023

875.76 -0.011

876.06 0.010

876.35 -0.005

876.65 0.014

876.95 0.012

>>>>>End Processed Spectral Data<<<<<
