# Supplementary material for: Two-Phase Bactericidal Mechanism of Silver Nanoparticles against Burkholderia pseudomallei
Source: PLoS One. 2016 Dec 15;11(12):e0168098. doi: 10.1371/journal.pone.0168098 (PMC5158019; doi:10.1371/journal.pone.0168098)
Supplement: S2 Data — (DOCX) [file pone.0168098.s002.docx]

**Raw Data**

**Fig 2.** **Killing kinetics of AgNPs against *B. pseudomallei****.* Bacterial suspensions strains NF10 (a) or 316a (b) were incubated with various concentrations of AgNPs and CAZ for 1, 2, 4, 6 and 24 h. Colonies were counted, and the bactericidal effects were defined as a ≥3-log reduction in colony-forming units (CFU)/mL compared to the initial inoculum. Data are the means of two independent experiments performed in triplicate. Short-term effects of AgNPs against *B. pseudomallei* NF10 and 316c were determined 0, 5, 30 and 60 min after exposure to AgNPs using a LIVE/DEAD^®^ BacLightTM bacteria viability kit by fluorescence microscopy (c).

**Raw data of Fig 2a.**

| **Times(h)** | **MIC of AgNPs** | **SD** | **MBC of AgNPs** | **SD** | **MIC of CAZ** | **SD** | **Negative**  **control** | **SD** |
| --- | --- | --- | --- | --- | --- | --- | --- | --- |
| 0  1  2  4  6  24 | 6.73471  1.33333  1  1  1  3.25938 | ±0.13744  ±0.5164  ±0  ±0  ±0  ±0.2158 | 6.80047  1  1  1  1  1 | ±0.03457  ±0  ±0  ±0  ±0  ±0 | 7.09668  6.35827  5.40137  4.51877  3.46007  3.8198 | ±0.07988  ±0.05957  ±0.15545  ±0.06452  ±0.13527  ±0.06452 | 6.66076  6.82571  7.61503  8.25938  8.85916  10.11148 | ±0.09093  ±0.13957  ±0.25185  ±0.2158  ±0.0795  ±0.05004 |

**Raw data of Fig 2b.**

| **Times(h)** | **MIC of AgNPs** | **SD** | **MBC of AgNPs** | **SD** | **MIC of CAZ** | **SD** | **Negative**  **control** | **SD** |
| --- | --- | --- | --- | --- | --- | --- | --- | --- |
| 0  1  2  4  6  24 | 6.95244  3.20069  1  1  2.46007  2.63436 | ±0.04336  ±0.15545  ±0  ±0  ±0.13527  ±0.31359 | 6.81572  1  1  1  1  1 | 0.09408  0  0  0  0  0 | 6.40137  6.25902  5.70012  3.40137  1  1 | ±0.15545  ±0.06711  ±0.54906  ±0.15545  ±0  ±0 | 6.49237  6.97476  7.20069  7.9246  8.66076  9.71308 | ±0.17836  ±0.1065  ±0.15545  ±0.11994  ±0.09093  ±0.01154 |

**Fig 3**. **Hemolytic activity of AgNPs**. Human erythrocytes were incubated in PBS with various concentrations of AgNPs for 1 hour at 37°C. The hemoglobin release was monitored using a microplate reader at the absorbance at 405 nm. Data are the means of two independent experiments performed in triplicate.

| **Concentration**  **(µg/ml)** | **% Hemolysis**  **of AgNPs** | **SD** | **% Hemolysis**  **of CAZ** | **SD** | **Triton X-10** |
| --- | --- | --- | --- | --- | --- |
| 2  4  8  16  32  64  128  256  512  1,024 | 2.612  2.74733  -0.13533  0.969  1.26767  0.424  4.23567  11.729  82.19433  85.58967 | ±0.95350  ±0.18103  ±0.77897  ±1.85566  ±2.24501  ±0.39969  ±1.38584  ±1.6893  ±1.19087  ±1.79156 | 0.52266  2.65233  2.51467  -1.74133  1.91433  2.227  3.05733  1.41133  0.44533  2.362 | ±0.68780  ±0.30055  ±0.49033  ±0.1379  ±0.30099  ±0.49381  ±0.3855  ±1.09522  ±0.1321  ±0.5301 | Not measured  Not measured  Not measured  Not measured  Not measured  Not measured  Not measured  Not measured  Not measured  100 |

**Fig 4.** **Effect of AgNP on bacterial viability and cell-surface charge of *B. pseudomallei***. *B. pseudomallei* NF10 (a) and 316c (b) was treated with AgNP concentrations of 8, 32, 64, 96, 128 and 256 µg/mL. Black squares corresponds to the percentage of viable bacterial cells in the presence of increasing AgNPs concentrations, whereas the zeta potential is indicated by the white squares. The dotted line indicates a neutral surface net charge, to highlight the AgNP concentration range at which both strains of *B. pseudomallei* exhibit surface neutrality and possible overcompensation is achieved. Data are the means of two independent experiments performed in triplicate.

**Raw data of figure 4a**

| **Concentration**  **(µg/ml)** | **% Bacterial Viability** | **SD** | **Zeta potential (mV)** | **SD** |
| --- | --- | --- | --- | --- |
| 0  8  32  64  96  128  256 | 100  29.44  19.64  0.01  0  0  0 | ±0  ±2.6  ±2.1  ±1  ±0.56  ±0  ±0 | -8.72  -5.47  -6.204  -7.16  -7.6  -7.23  -4.07 | ±0.183  ±0.168  ±0.139  ±0.155  ±0.19  ±0.196  ±0.284 |

**Fig 5. Kinetics of silver ion release on *B.pseudomallei* 316c.** *B.pseudomallei* 316c was treated with AgNPs and AgNO_3_ at 48 µg/mL for 0, 0.5,1,4 and 24 h at 37°C. The silver ion release was measured using ICP-OES technique. The killing kinetic was measured using dilution plate count method. Both of data are the mean of two experiments performed in triplicate.

**Raw data of Fig 5a.**

| Times (h) | Ag ion releasing (µg/mL) of AgNP+Cell | SD | Log CFU/mL of AgNPs+Cell | SD |
| --- | --- | --- | --- | --- |
| 0 | 0.0013 | ±0.005 | 7.477 | ±0.045 |
| 0.5 | 0.1917 | ±0.0029 | 0 | ±0 |
| 1 | 0.1933 | ±0.0248 | 0 | ±0 |
| 4 | 0.67 | ±0.0329 | 0 | ±0 |
| 24 | 0.706 | ±0.0336 | 2.397 | ±0.067 |

**Raw data of Fig 5b.**

| Times (h) | Ag ion releasing (µg/mL) of AgNP+Cell | SD | Log CFU/mL of AgNPs+Cell | SD |
| --- | --- | --- | --- | --- |
| 0 | 0.0013 | ±0.005 | 7.477 | ±0.045 |
| 0.5 | 17.4327 | ±0.068 | 0 | ±0 |
| 1 | 10.67 | ±0.1521 | 0 | ±0 |
| 4 | 8.591 | ±0.1253 | 0 | ±0 |
| 24 | 2.409 | ±0.0201 | 0 | ±0 |

**Fig 6.** **Kinetics of ROS induction in *B. pseudomallei*** **after exposure to AgNPs.** Kinetics of induction of ROS in *B. pseudomallei* NF10 (a) and 316c (b) and accumulated ROS at 4 hrs (c) were followed by DCF fluorescence. All bacterial cells were treated with various concentrations of AgNPs: 0 μg/mL, 1/5 MIC, 1/2 MIC, MIC and MBC of AgNPs. 30% H_2_O_2_ was used as a positive control. The overall P-value was determined using one-way ANOVA. * indicates a P value of <0.001 in comparison to bacterial cells treated with deionize water (0 μg/mL), δ indicates a P value of < 0.007 in comparison of MIC with MBC treated condition. Data represent the means standard errors of the means for triplicate samples. Results shown are representative of two independent experiments.

**Raw data of Fig 6a.**

| **Time (h)** | **0** | | **1** | | **2** | | **4** | |
| --- | --- | --- | --- | --- | --- | --- | --- | --- |
|  | **FI _DCF_** | **SD** | **FI _DCF_** | **SD** | **FI _DCF_** | **SD** | **FI _DCF_** | **SD** |
| 0 | 1.875 | ±0.14323 | 2.833 | ±0.07829 | 3.352 | ±1.07996 | 4.309 | ±0.24027 |
| 1/5 MIC | 2.001 | ±0.11605 | 5.633 | ±0.39292 | 3.506 | ±0.18943 | 5.007 | ±0.83168 |
| ½ MIC | 2.182 | ±0.15578 | 3.163 | ±0.05565 | 3.883 | ±0.16066 | 4.536 | ±0.47718 |
| MIC | 1.882 | ±0.11605 | 3.604 | ±0.04644 | 4.472 | ±0.40384 | 5.98 | ±0.88807 |
| MBC | 1.95 | ±0.15578 | 4.24 | ±0.17327 | 6.71 | ±0.34271 | 9.921 | ±1.32122 |
| H_2_O_2_ | 2.18 | ±0.11605 | 47.927 | ±1.88652 | 40.721 | ±1.5015 | 21.749 | ±0.68496 |

** FI : Fluorescence Intensity

**Raw data of Fig 6b.**

| **Time (h)** | **0** | | **1** | | **2** | | **4** | |
| --- | --- | --- | --- | --- | --- | --- | --- | --- |
|  | **FI _DCF_** | **SD** | **FI _DCF_** | **SD** | **FI _DCF_** | **SD** | **FI _DCF_** | **SD** |
| 0 | 1.801 | ±0.03367 | 3.212 | ±0.34036 | 3.903 | ±1.30532 | 4.667 | ±0.79061 |
| 1/5 MIC | 2.826 | ±0.11605 | 3.463 | ±0.10362 | 4.8 | ±0.24024 | 5.948 | ±1.93236 |
| ½ MIC | 2.774 | ±0.15578 | 3.4 | ±0.05452 | 4.22 | ±0.15325 | 5.272 | ±1.69486 |
| MIC | 2.004 | ±0.11605 | 3.533 | ±0.15799 | 6.347 | ±0.20585 | 12.824 | ±4.60176 |
| MBC | 1.893 | ±0.15578 | 5.904 | ±0.08984 | 10.205 | ±0.38398 | 19.11 | ±3.90294 |
| H_2_O_2_ | 1.967 | ±0.11605 | 58.944 | ±2.06023 | 51.961 | ±2.4515 | 28.432 | ±1.49155 |

** FI : Fluorescence Intensity

**Raw data of Fig. 6c**

| **Isolates of  *B. pseudomallei*** | ***B. pseudomallei* NF10** | | ***B. pseudomallei* 316C** | | **E. coli O157:H7** | |
| --- | --- | --- | --- | --- | --- | --- |
|  | **FI _DCF_** | **SD** | **FI _DCF_** | **SD** | **FI _DCF_** | **SD** |
| 0 | 2.8705 | ±0.02483 | 3.007 | ±0.16512 | 2.826 | ±0.05774 |
| 1/5 MIC | 9.852 | ±0.67204 | 10.4335 | ±0.65991 | 9.235 | ±0.04734 |
| ½ MIC | 9.745 | ±0.31408 | 10.605 | ±0.35911 | 9.9035 | ±0.19572 |
| MIC | 11.471 | ±0.63393 | 11.7455 | ±0.40819 | 12.4475 | ±0.15415 |
| MBC | 18.3955 | ±0.19919 | 19.2045 | ±0.59178 | 19.877 | ±0.38913 |
| 30% H_2_O_2_ | 90.2635 | ±3.48084 | 85.6185 | ±0.84235 | 92.3765 | ±3.691 |
