# Supplementary material for: Two-Phase Bactericidal Mechanism of Silver Nanoparticles against Burkholderia pseudomallei
Source: PLoS One. 2016 Dec 15;11(12):e0168098. doi: 10.1371/journal.pone.0168098 (PMC5158019; doi:10.1371/journal.pone.0168098)
Supplement: S3 Data — (RTF) [file pone.0168098.s003.rtf]

Oneway


ONEWAY VAR00004 BY VAR00001   /MISSING ANALYSIS   /POSTHOC=LSD ALPHA(0.001).


Notes	
	Output Created	10-ÁÔ.Â.-2559, 10 ¹ÒÌÔ¡Ò 37 ¹Ò·Õ	
	Comments		
Input	Active Dataset	DataSet0	
	Filter	<none>	
	Weight	<none>	
	Split File	<none>	
	N of Rows in Working Data File	18	
Missing Value Handling	Definition of Missing	User-defined missing values are treated as missing.	
	Cases Used	Statistics for each analysis are based on cases with no missing data for any variable in the analysis.	
	Syntax	ONEWAY VAR00004 BY VAR00001
  /MISSING ANALYSIS
  /POSTHOC=LSD ALPHA(0.001).
	
Resources	Processor Time	0:00:00.000	
	Elapsed Time	0:00:00.027	

B.pseudomallei(nf10)


ANOVA	
VAR00004	
	Sum of Squares	df	Mean Square	F	Sig.	
Between Groups	16750.693	5	3350.139	2796.598	.000	
Within Groups	14.375	12	1.198			
Total	16765.068	17				


Post Hoc Tests


Multiple Comparisons	
VAR00004
LSD	
(I) VAR00001	(J) VAR00001		
		Mean Difference (I-J)	Std. Error	Sig.	
1	2	-6.9653333*	.8936568	.000	
	3	-6.6225000*	.8936568	.000	
	4	-8.6393667*	.8936568	.000	
	5	-15.4972333*	.8936568	.000	
	6	-88.4968333*	.8936568	.000	
2	1	6.9653333*	.8936568	.000	
	3	.3428333	.8936568	.708	
	4	-1.6740333	.8936568	.086	
	5	-8.5319000*	.8936568	.000	
	6	-81.5315000*	.8936568	.000	
3	1	6.6225000*	.8936568	.000	
	2	-.3428333	.8936568	.708	
	4	-2.0168667	.8936568	.043	
	5	-8.8747333*	.8936568	.000	
	6	-81.8743333*	.8936568	.000	
4	1	8.6393667*	.8936568	.000	
	2	1.6740333	.8936568	.086	
	3	2.0168667	.8936568	.043	
	5	-6.8578667*	.8936568	.000	
	6	-79.8574667*	.8936568	.000	
5	1	15.4972333*	.8936568	.000	
	2	8.5319000*	.8936568	.000	
	3	8.8747333*	.8936568	.000	
	4	6.8578667*	.8936568	.000	
	6	-72.9996000*	.8936568	.000	
6	1	88.4968333*	.8936568	.000	
	2	81.5315000*	.8936568	.000	
	3	81.8743333*	.8936568	.000	
	4	79.8574667*	.8936568	.000	
	5	72.9996000*	.8936568	.000	
*. The mean difference is significant at the 0.001 level.	

Multiple Comparisons	
VAR00004
LSD	
(I) VAR00001	(J) VAR00001	99.9% Confidence Interval	
		Lower Bound	Upper Bound	
1	2	-10.823957	-3.106710	
	3	-10.481123	-2.763877	
	4	-12.497990	-4.780743	
	5	-19.355857	-11.638610	
	6	-92.355457	-84.638210	
2	1	3.106710	10.823957	
	3	-3.515790	4.201457	
	4	-5.532657	2.184590	
	5	-12.390523	-4.673277	
	6	-85.390123	-77.672877	
3	1	2.763877	10.481123	
	2	-4.201457	3.515790	
	4	-5.875490	1.841757	
	5	-12.733357	-5.016110	
	6	-85.732957	-78.015710	
4	1	4.780743	12.497990	
	2	-2.184590	5.532657	
	3	-1.841757	5.875490	
	5	-10.716490	-2.999243	
	6	-83.716090	-75.998843	
5	1	11.638610	19.355857	
	2	4.673277	12.390523	
	3	5.016110	12.733357	
	4	2.999243	10.716490	
	6	-76.858223	-69.140977	
6	1	84.638210	92.355457	
	2	77.672877	85.390123	
	3	78.015710	85.732957	
	4	75.998843	83.716090	
	5	69.140977	76.858223	
	


ONEWAY VAR00004 BY VAR00001   /MISSING ANALYSIS   /POSTHOC=LSD ALPHA(0.001).


Oneway


Notes	
	Output Created	10-ÁÔ.Â.-2559, 10 ¹ÒÌÔ¡Ò 43 ¹Ò·Õ	
	Comments		
Input	Active Dataset	DataSet0	
	Filter	<none>	
	Weight	<none>	
	Split File	<none>	
	N of Rows in Working Data File	18	
Missing Value Handling	Definition of Missing	User-defined missing values are treated as missing.	
	Cases Used	Statistics for each analysis are based on cases with no missing data for any variable in the analysis.	
	Syntax	ONEWAY VAR00004 BY VAR00001
  /MISSING ANALYSIS
  /POSTHOC=LSD ALPHA(0.001).
	
Resources	Processor Time	0:00:00.000	
	Elapsed Time	0:00:00.010	

B.pseudomallei 316c


ANOVA	
VAR00004	
	Sum of Squares	df	Mean Square	F	Sig.	
Between Groups	14329.297	5	2865.859	13150.095	.000	
Within Groups	2.615	12	.218			
Total	14331.913	17				


Post Hoc Tests


Multiple Comparisons	
VAR00004
LSD	
(I) VAR00001	(J) VAR00001		
		Mean Difference (I-J)	Std. Error	Sig.	
1	2	-7.3861000*	.3811688	.000	
	3	-7.5700667*	.3811688	.000	
	4	-8.7287000*	.3811688	.000	
	5	-16.2426667*	.3811688	.000	
	6	-82.6318667*	.3811688	.000	
2	1	7.3861000*	.3811688	.000	
	3	-.1839667	.3811688	.638	
	4	-1.3426000	.3811688	.004	
	5	-8.8565667*	.3811688	.000	
	6	-75.2457667*	.3811688	.000	
3	1	7.5700667*	.3811688	.000	
	2	.1839667	.3811688	.638	
	4	-1.1586333	.3811688	.010	
	5	-8.6726000*	.3811688	.000	
	6	-75.0618000*	.3811688	.000	
4	1	8.7287000*	.3811688	.000	
	2	1.3426000	.3811688	.004	
	3	1.1586333	.3811688	.010	
	5	-7.5139667*	.3811688	.000	
	6	-73.9031667*	.3811688	.000	
5	1	16.2426667*	.3811688	.000	
	2	8.8565667*	.3811688	.000	
	3	8.6726000*	.3811688	.000	
	4	7.5139667*	.3811688	.000	
	6	-66.3892000*	.3811688	.000	
6	1	82.6318667*	.3811688	.000	
	2	75.2457667*	.3811688	.000	
	3	75.0618000*	.3811688	.000	
	4	73.9031667*	.3811688	.000	
	5	66.3892000*	.3811688	.000	
*. The mean difference is significant at the 0.001 level.	

Multiple Comparisons	
VAR00004
LSD	
(I) VAR00001	(J) VAR00001	99.9% Confidence Interval	
		Lower Bound	Upper Bound	
1	2	-9.031907	-5.740293	
	3	-9.215874	-5.924259	
	4	-10.374507	-7.082893	
	5	-17.888474	-14.596859	
	6	-84.277674	-80.986059	
2	1	5.740293	9.031907	
	3	-1.829774	1.461841	
	4	-2.988407	.303207	
	5	-10.502374	-7.210759	
	6	-76.891574	-73.599959	
3	1	5.924259	9.215874	
	2	-1.461841	1.829774	
	4	-2.804441	.487174	
	5	-10.318407	-7.026793	
	6	-76.707607	-73.415993	
4	1	7.082893	10.374507	
	2	-.303207	2.988407	
	3	-.487174	2.804441	
	5	-9.159774	-5.868159	
	6	-75.548974	-72.257359	
5	1	14.596859	17.888474	
	2	7.210759	10.502374	
	3	7.026793	10.318407	
	4	5.868159	9.159774	
	6	-68.035007	-64.743393	
6	1	80.986059	84.277674	
	2	73.599959	76.891574	
	3	73.415993	76.707607	
	4	72.257359	75.548974	
	5	64.743393	68.035007	
	


ONEWAY VAR00004 BY VAR00001   /MISSING ANALYSIS   /POSTHOC=LSD ALPHA(0.001).


Oneway


Notes	
	Output Created	10-ÁÔ.Â.-2559, 10 ¹ÒÌÔ¡Ò 48 ¹Ò·Õ	
	Comments		
Input	Active Dataset	DataSet0	
	Filter	<none>	
	Weight	<none>	
	Split File	<none>	
	N of Rows in Working Data File	18	
Missing Value Handling	Definition of Missing	User-defined missing values are treated as missing.	
	Cases Used	Statistics for each analysis are based on cases with no missing data for any variable in the analysis.	
	Syntax	ONEWAY VAR00004 BY VAR00001
  /MISSING ANALYSIS
  /POSTHOC=LSD ALPHA(0.001).
	
Resources	Processor Time	0:00:00.016	
	Elapsed Time	0:00:00.005	

E.coli


ANOVA	
VAR00004	
	Sum of Squares	df	Mean Square	F	Sig.	
Between Groups	17142.614	5	3428.523	2256.674	.000	
Within Groups	18.231	12	1.519			
Total	17160.845	17				


Post Hoc Tests


Multiple Comparisons	
VAR00004
LSD	
(I) VAR00001	(J) VAR00001		
		Mean Difference (I-J)	Std. Error	Sig.	
1	2	-6.2727667*	1.0064067	.000	
	3	-7.1239000*	1.0064067	.000	
	4	-9.6339333*	1.0064067	.000	
	5	-17.3744333*	1.0064067	.000	
	6	-89.7334667*	1.0064067	.000	
2	1	6.2727667*	1.0064067	.000	
	3	-.8511333	1.0064067	.414	
	4	-3.3611667	1.0064067	.006	
	5	-11.1016667*	1.0064067	.000	
	6	-83.4607000*	1.0064067	.000	
3	1	7.1239000*	1.0064067	.000	
	2	.8511333	1.0064067	.414	
	4	-2.5100333	1.0064067	.028	
	5	-10.2505333*	1.0064067	.000	
	6	-82.6095667*	1.0064067	.000	
4	1	9.6339333*	1.0064067	.000	
	2	3.3611667	1.0064067	.006	
	3	2.5100333	1.0064067	.028	
	5	-7.7405000*	1.0064067	.000	
	6	-80.0995333*	1.0064067	.000	
5	1	17.3744333*	1.0064067	.000	
	2	11.1016667*	1.0064067	.000	
	3	10.2505333*	1.0064067	.000	
	4	7.7405000*	1.0064067	.000	
	6	-72.3590333*	1.0064067	.000	
6	1	89.7334667*	1.0064067	.000	
	2	83.4607000*	1.0064067	.000	
	3	82.6095667*	1.0064067	.000	
	4	80.0995333*	1.0064067	.000	
	5	72.3590333*	1.0064067	.000	
*. The mean difference is significant at the 0.001 level.	

Multiple Comparisons	
VAR00004
LSD	
(I) VAR00001	(J) VAR00001	99.9% Confidence Interval	
		Lower Bound	Upper Bound	
1	2	-10.618221	-1.927313	
	3	-11.469354	-2.778446	
	4	-13.979387	-5.288479	
	5	-21.719887	-13.028979	
	6	-94.078921	-85.388013	
2	1	1.927313	10.618221	
	3	-5.196587	3.494321	
	4	-7.706621	.984287	
	5	-15.447121	-6.756213	
	6	-87.806154	-79.115246	
3	1	2.778446	11.469354	
	2	-3.494321	5.196587	
	4	-6.855487	1.835421	
	5	-14.595987	-5.905079	
	6	-86.955021	-78.264113	
4	1	5.288479	13.979387	
	2	-.984287	7.706621	
	3	-1.835421	6.855487	
	5	-12.085954	-3.395046	
	6	-84.444987	-75.754079	
5	1	13.028979	21.719887	
	2	6.756213	15.447121	
	3	5.905079	14.595987	
	4	3.395046	12.085954	
	6	-76.704487	-68.013579	
6	1	85.388013	94.078921	
	2	79.115246	87.806154	
	3	78.264113	86.955021	
	4	75.754079	84.444987	
	5	68.013579	76.704487	
	


Notes	
	Output Created	10-ÁÔ.Â.-2559, 11 ¹ÒÌÔ¡Ò 22 ¹Ò·Õ	
	Comments		
Input	Active Dataset	DataSet0	
	Filter	<none>	
	Weight	<none>	
	Split File	<none>	
	N of Rows in Working Data File	18	
Missing Value Handling	Definition of Missing	User-defined missing values are treated as missing.	
	Cases Used	Statistics for each analysis are based on cases with no missing data for any variable in the analysis.	
	Syntax	ONEWAY VAR00004 BY VAR00001
  /MISSING ANALYSIS
  /POSTHOC=LSD ALPHA(0.05).
	
Resources	Processor Time	0:00:00.000	
	Elapsed Time	0:00:00.004	


Notes	
	Output Created	10-ÁÔ.Â.-2559, 11 ¹ÒÌÔ¡Ò 25 ¹Ò·Õ	
	Comments		
Input	Active Dataset	DataSet0	
	Filter	<none>	
	Weight	<none>	
	Split File	<none>	
	N of Rows in Working Data File	18	
Missing Value Handling	Definition of Missing	User-defined missing values are treated as missing.	
	Cases Used	Statistics for each analysis are based on cases with no missing data for any variable in the analysis.	
	Syntax	ONEWAY VAR00004 BY VAR00001
  /MISSING ANALYSIS
  /POSTHOC=LSD ALPHA(0.05).
	
Resources	Processor Time	0:00:00.016	
	Elapsed Time	0:00:00.006	


Notes	
	Output Created	10-ÁÔ.Â.-2559, 11 ¹ÒÌÔ¡Ò 31 ¹Ò·Õ	
	Comments		
Input	Active Dataset	DataSet0	
	Filter	<none>	
	Weight	<none>	
	Split File	<none>	
	N of Rows in Working Data File	18	
Missing Value Handling	Definition of Missing	User-defined missing values are treated as missing.	
	Cases Used	Statistics for each analysis are based on cases with no missing data for any variable in the analysis.	
	Syntax	ONEWAY VAR00004 BY VAR00001
  /MISSING ANALYSIS.
	
Resources	Processor Time	0:00:00.000	
	Elapsed Time	0:00:00.004	


ONEWAY VAR00004 BY VAR00001   /MISSING ANALYSIS.


Oneway


Notes	
	Output Created	10-ÁÔ.Â.-2559, 11 ¹ÒÌÔ¡Ò 33 ¹Ò·Õ	
	Comments		
Input	Active Dataset	DataSet0	
	Filter	<none>	
	Weight	<none>	
	Split File	<none>	
	N of Rows in Working Data File	18	
Missing Value Handling	Definition of Missing	User-defined missing values are treated as missing.	
	Cases Used	Statistics for each analysis are based on cases with no missing data for any variable in the analysis.	
	Syntax	ONEWAY VAR00004 BY VAR00001
  /MISSING ANALYSIS.
	
Resources	Processor Time	0:00:00.000	
	Elapsed Time	0:00:00.007	

B.pseudomallei nf10 (MIC compared MBC)


ANOVA	
VAR00004	
	Sum of Squares	df	Mean Square	F	Sig.	
Between Groups	70.546	1	70.546	414.730	.000	
Within Groups	.680	4	.170			
Total	71.226	5				


ONEWAY VAR00004 BY VAR00001   /MISSING ANALYSIS.


Oneway


Notes	
	Output Created	10-ÁÔ.Â.-2559, 11 ¹ÒÌÔ¡Ò 35 ¹Ò·Õ	
	Comments		
Input	Active Dataset	DataSet0	
	Filter	<none>	
	Weight	<none>	
	Split File	<none>	
	N of Rows in Working Data File	18	
Missing Value Handling	Definition of Missing	User-defined missing values are treated as missing.	
	Cases Used	Statistics for each analysis are based on cases with no missing data for any variable in the analysis.	
	Syntax	ONEWAY VAR00004 BY VAR00001
  /MISSING ANALYSIS.
	
Resources	Processor Time	0:00:00.000	
	Elapsed Time	0:00:00.007	

B. pseudomallei 316c (MIC compared MBC)


ANOVA	
VAR00004	
	Sum of Squares	df	Mean Square	F	Sig.	
Between Groups	84.690	1	84.690	493.454	.000	
Within Groups	.687	4	.172			
Total	85.376	5				


ONEWAY VAR00004 BY VAR00001   /MISSING ANALYSIS.


Oneway


Notes	
	Output Created	10-ÁÔ.Â.-2559, 11 ¹ÒÌÔ¡Ò 38 ¹Ò·Õ	
	Comments		
Input	Active Dataset	DataSet0	
	Filter	<none>	
	Weight	<none>	
	Split File	<none>	
	N of Rows in Working Data File	18	
Missing Value Handling	Definition of Missing	User-defined missing values are treated as missing.	
	Cases Used	Statistics for each analysis are based on cases with no missing data for any variable in the analysis.	
	Syntax	ONEWAY VAR00004 BY VAR00001
  /MISSING ANALYSIS.
	
Resources	Processor Time	0:00:00.000	
	Elapsed Time	0:00:00.005	

E.coli


ANOVA	
VAR00004	
	Sum of Squares	df	Mean Square	F	Sig.	
Between Groups	89.873	1	89.873	213.028	.000	
Within Groups	1.688	4	.422			
Total	91.561	5				
